# Cell Survival Is Regulated via SOX9/BCL2L1 Axis in HCT-116 Colorectal Cancer Cell Line

**DOI:** 10.1155/2020/5701527

**Published:** 2020-04-29

**Authors:** Erik Lizárraga-Verdugo, Erika Ruiz-García, César López-Camarillo, Mercedes Bermúdez, Mariana Avendaño-Félix, Rosalío Ramos-Payán, Geovanni Romero-Quintana, Alfredo Ayala-Ham, Carlos Villegas-Mercado, Carlos Pérez-Plasencia, Maribel Aguilar-Medina

**Affiliations:** ^1^Facultad de Ciencias Químico Biológicas, Universidad Autónoma de Sinaloa, Culiacán, Sinaloa, Mexico; ^2^Instituto Nacional de Cancerología, CDMX, Mexico; ^3^Posgrado en Ciencias Genómicas, Universidad Autónoma de La Ciudad de México, CDMX, Mexico; ^4^Facultad de Odontología, Universidad Autónoma de Sinaloa, Culiacán, Sinaloa, Mexico

## Abstract

Colorectal cancer (CRC) is one of the most frequent types of malignancies and one of the major causes of cancer-related death worldwide. Sex-determining region Y (SRY)-box 9 protein (SOX9) is a member of the SOX family of transcription factors which are involved in the regulation of differentiation and development. Recently, several reports suggest an important role of SOX9 in tumorigenesis since its overexpression correlates with tumor progression and poor outcome in several types of cancer; however, its role in CRC is not clear until now. Therefore, in this work, we searched for novel SOX9-regulated genes involved in cell survival of CRC. We silenced SOX9 in the poorly differentiated HCT-116 cell line, using a specific siRNA, to identify differential expressed genes by DNA microarrays and analyzed the role or candidate genes in apoptosis and autophagy. Transcriptome analysis showed that diverse cellular pathways, associated with CRC carcinogenesis such as Wnt/*β*-catenin, MAPK, TGF-*β*, and mTOR, were modulated after SOX9 silencing. Interestingly, we found that SOX9 silencing promotes downregulation of BCL2L1 and overexpression of CASP3, proteins related to apoptosis, which was further confirmed in SW-480, a moderated-differentiated cell line, but not in HT-29, well-differentiated cell line. Moreover, inhibition of BCL2L1 by ABT-737 (BH3 mimetic) in SOX9-silenced HCT-116 cells resulted in an increased apoptosis percentage. However, downregulation of BCL2L1 was not enough to induce autophagy. This is the first report, suggesting that cell survival in poorly and moderated-differentiated CRC cells lines is regulated by SOX9/BCL2L1 axis, but not in well-differentiated cell lines.

## 1. Introduction

Colorectal cancer (CRC) is a multifactorial disease that results from lifestyle, genetic, and environmental factors [1], and it is considered as one of the major causes of cancer-related death worldwide [2]. Intestinal epithelium exhibits a high turnover rate, which favors carcinogenesis [3]; in contrast, stem cells from intestinal crypts are responsible of tissue maintaining and homeostasis since they show a low rate of cell death, a process regulated by programmed cell death [4] through different mechanisms such as apoptosis, autophagy, and necroptosis [5]. Programmed cell death resistance is considered as a hallmark of cancer [6].

There are two basic signaling pathways that regulate apoptosis: extrinsic and intrinsic [5, 7]. The extrinsic pathway is stimulated by interaction of ligands to cell surface-exposed death receptors that culminates in caspase-8 activation, for example, the tumor necrosis factor receptors (TNFRs) and their respective protein TNF family ligand, among others [8]. On the contrary, the intrinsic pathway is mitochondria-dependent mediated by intracellular signals in response to different stress conditions including chemotherapeutic agents, DNA damage, growth factor deprivation, and oxidative stress, among others [9]. The activation of intrinsic apoptosis pathway drives to apoptosome formation, which is composed of procaspase-9, apoptotic protease-activating factor (Apaf-1), and cytochrome c, that concludes in caspase-9 activation [5]. This process is led by Bcl-2 family members' proteins that can be divided into two groups: proapoptotic or antisurvival (Bax, Bak, Bid, Bad, and Bok) and antiapoptotic or prosurvival (Bcl-2, Bcl-w, and Bcl-xL) [10]. Bax drives to apoptosome formation and caspase-3 activation, promoting apoptosis and has been reported that Bcl-xL, encoded by BCL2L1 (BCL2-like 1) gene [11], is able to stabilize the mitochondrial localization of Bax, maintaining it in an inactive state [12], which is a mechanism implicated in cell survival in different tumor types [13].

Several signaling pathways have key roles in the regulation of cell survival within tumor tissues, for instance, Wnt/*β*-catenin signaling pathway, which in turn is regulated by SOX9 through two main mechanisms: *ββ*-catenin degradation and transcriptional activity inhibition [14–16]. SOX9 is a member of SOX (SRY (sex-determining region Y)-related high mobility group (HMG) box) family and works as a transcription factor that plays a central role in the development and differentiation of multiple cell lineage [17]. Overexpression and mutation of SOX9 increases cell proliferation, invasiveness, and metastasis in several types of cancer [18–21]; nonetheless, little is known about SOX9 and its importance in cell survival in CRC. Therefore, the aim of this study was to identify novel genes regulated by SOX9 and their role in cell survival in CRC.

## 2. Materials and Methods

### 2.1. Cell Culture

The human colorectal cancer cell lines HCT-116, SW-480, Caco-2, HT-29, and nontumorigenic cell line CCD-18Co were obtained from American Type Culture Collection (ATCC). HCT-116 and HT29 were cultured in DMEM/F12 (Gibco) supplemented with 10% fetal bovine serum (FBS) and 1% penicillin/streptomycin. SW-480 was cultured in RPMI 1640 (Gibco) supplemented with 15% FBS and 1% penicillin/streptomycin. CCD-18Co and Caco-2 were grown in DMEM/F12 supplemented with 10% FBS, 1% penicillin/streptomycin, and 1% nonessential amino acids (NEAA). All cell lines were incubated at 37°C with 5% CO_2_ in a humidified atmosphere.

### 2.2. SOX9 Silencing

HCT-116 cell line was seeded at a density of 3 × 10^5^ cells per well into a 6-well plate, and after 70% of confluence was reached, cells were transfected with 30 nM of Silencer Select siRNA (ID: s532658, Thermo Fisher Scientific) specific to SOX9, using lipofectamine RNAiMAX reagent (Invitrogen) according to the manufacturer's protocol. SOX9 silencing confirmation was assessed by RT-qPCR at 24 h posttransfection.

### 2.3. RNA Isolation and RT-qPCR

Total RNA was isolated by TRIzol Reagent (Invitrogen) method following the manufacturer's instructions. cDNA synthesis was performed with 2 *μ*g of total RNA using High-Capacity cDNA Reverse Transcription Kit (Thermo Fisher Scientific). Real-time PCR was performed using TaqMan Gene Expression Assays (Applied Biosystems) and TaqMan™ Universal PCR Master Mix (Thermo Fisher Scientific) for SOX9 (Hs00165814_m1), BCL2L1 (Hs00236329_m1), and CASP3 (Hs00234387_m1). The mixtures were incubated at 50°C for 2 min, 95°C 10 min, followed by 40 cycles of 95°C for 15 s and 60°C for 1 min. Gene expression was measured in triplicate, and data were analyzed using 2^−ΔΔCT^ method. ACTB (Hs01060665_g1) was used as endogenous control.

### 2.4. Microarray Hybridization, Detection, and Preprocessing

Whole-genome transcriptome analysis was performed using Clariom D arrays (Affymetrix GeneChip) following manufacturer's instructions. In brief, the Affymetrix GeneChip WT Pico Kit was used for cDNA preparation and biotin labeling. cRNA was purified using an Affymetrix magnetic bead protocol. The Affymetrix GeneChip™ Hybridization, Wash, and Stain Kit was used for array processing. Arrays were incubated for 16 h in an Affymetrix GeneChip 645 hybridization oven at 45°C with rotation at 60 rpm. Fluorescence was amplified by adding biotinylated antistreptavidin and an additional aliquot of streptavidin-phycoerythrin stain. A confocal scanner (Affymetrix GeneChip Scanner 3000 7G plus) was used to collect the fluorescence signal at 3 *μ*m resolution after excitation at 570 nm. The average signal from two sequential scans was calculated for each microarray.

### 2.5. Microarray Data Analysis

Microarray data analysis was performed using Partek Genomic Suite v8.0. All samples were normalized with Robust Multiarray Average (RMA) [22], which includes background correction, normalization, and calculation of expression values. Differential expression analysis was performed using ANOVA one way. We selected differentially expressed (DE) genes between groups based on a fold-change of 2 in absolute value, and Benjamini and Hochberg false discovery rate [23] was applied for multiple hypotheses testing, and the genes with an adjusted *p* < 0.01 were accepted. The enrichment analysis with DAVID (Database for Annotation, Visualization, and Integrated Discovery) [24, 25], and Partek Genomic Suite v8.0 was performed on each list of selected genes. Partek Genomic Suite was also used for pathway analysis.

### 2.6. *In Silico* Analysis

Dataset from The Cancer Genome Atlas (TCGA) was queried and analyzed using the Gene Expression Profiling and Interactive Analyses (GEPIA) [26] platform (http://gepia.cancer-pku.cn/). A total of 275 CRC tissues were included and compared with 349 normal adjacent tissues in order to compare SOX9 expression. Finally, Partek Genomic Suite was also used for pathway analysis, and interactome analysis was developed in String (https://string-db.org/cgi/network.pl?taskId=Y8RKNUbzndpT) in order to identify association between DE genes.

### 2.7. Flow Cytometry Apoptosis Analysis

A total of 90,000 cells were seeded in a 24-wells plate and incubated at 37°C for 24 h, and then the fresh medium containing 5 *μ*M ABT-737 (Abcam, ab141336), a BH3 mimetic, was added for 48 h to inhibit BCL2L1 function. According to the recommended protocol, the apoptosis rates were analyzed by Annexin-V-PE and 7-AAD double-staining method based on the PE Annexin V Apoptosis Detection Kit I (BD Pharmingen, 559763) through flow cytometry using a BD Accuri C6 (BD Biosciences, San Jose, CA, USA). Data were analyzed using FlowJo v.10.

### 2.8. Immunofluorescence Analysis

80,000 cells were grown in round-glass cover slips into a 24-well plate. Immunofluorescence was performed after 48 h posttransfection. In brief, cells were washed with cold 1X PBS and fixated with 4% *p*-formaldehyde for 10 min. Samples were washed three times with 1X PBS and permeabilized with 0.1% Triton X-100 for 10 min. After permeabilization, samples were washed and blocked with 22.52 mg/mL glycine and 1% bovine serum albumin. Samples were hybridized with 1 : 200 rabbit anti-human SOX9 (Abcam ab185966) or 1 : 500 rabbit anti-human LC3B (Abcam ab51520) for 1 h at room temperature and then washed and incubated with 1 : 1,000 secondary antibody donkey anti-rabbit IgG Alexa 647 (Abcam ab150075) for 1 h at room temperature. Anti-PGA3 antibody (7G3) (Abcam ab50123) and anti-GAPDH antibody (Abcam ab9485) were used as an isotype control and loading control, respectively. Finally, samples were washed and mounted with Fluoroshield with DAPI (Sigma). Images were obtained at a 40x magnification in a Leica LSCM (TCS SP8 Leica Microsystems). Mean of fluorescent intensity was calculated by choosing five ROIs in each experiment using Leica Application Suite *X*.

### 2.9. Statistical Analysis

Experiments were performed three times by triplicate, and results were represented as mean ± S.D. The student's *t*-test was used when comparing two parametric variables; ANOVA and multiple comparisons Tukey's test were used for 3 or more parametric variables. *p* < 0.05 was considered as statistically significant.

## 3. Results and Discussion

### 3.1. SOX9 Is Overexpressed in CRC Tumors and Cell Lines

**Figure 1:**
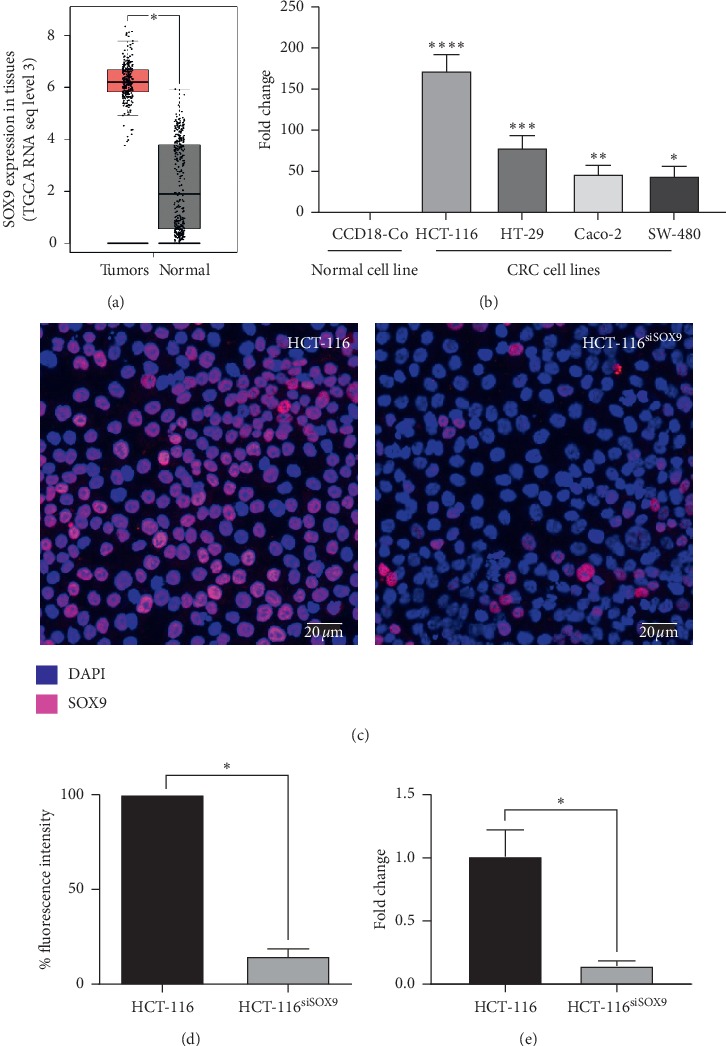
SOX9 is overexpressed in tumors and CRC cells lines. (a) TCGA datasets in silico analysis showed that SOX9 is overexpressed in colon cancer tissues in comparison with adjacent normal samples (^*∗*^*p* < 0.001). (b) Quantitative RT-qPCR showed that SOX9 is overexpressed in all studied CRC cell lines in comparison with the nontumorigenic CCD-18Co cell line (all *p* < 0.001). (c) Immunofluorescence assays showed that nuclear SOX9 expression is highly diminished in HCT-116 SOX9-silenced cells. (d) Fluorescence intensity mean in HCT-116 SOX9-silenced cells compared with control (^*∗*^*p*=0.001). (e) RT-qPCR analysis confirmed SOX9 silencing in HCT-116 cells (^*∗*^*p*=0.002).

*In silico* analysis of 275 CRC tumor tissues, of patients with colon adenocarcinoma from TCGA database, showed a higher SOX9 expression levels (LogFch∼3.0) in comparison with 349 healthy adjacent tissues (*p* < 0.01) (Figure 1(a)). These results show the same pattern when compared with other types of cancer such as renal cell carcinoma (RCC). Besides, overexpression of SOX9 is related to clinicopathological characteristics, such as the advanced pathological grade and clinical stage. Also, SOX9 is an independent predictor factor for the survival of RCC patients in the TCGA dataset [27].

In our study, we found that SOX9 is overexpressed in HCT-116 (*p*=0.0061), HT-29 (*p*=0.0151), Caco-2 (*p*=0.0269), and SW-480 (*p*=0.0405) CRC cell lines (Figure 1(b)). In particular, the poorly differentiated HCT-116 showed the highest expression levels (LogFch = 173.56); thus, it was selected for downstream analysis. Interestingly, SOX9 expression has been related with lower overall survival, tumor size, tumor progression, metastasis, and cancer cell plasticity in CRC samples at advanced stages [28–30].

### 3.2. SOX9 Silencing Induces Important Changes in Transcriptome Expression of HCT-116 Cells

SOX9 protein level was decreased 84.4% (*p*=0.001) in transfected HCT-116 (HCT-116^siSOX9^) (Figures 1(c) and 1(d)). Comparable results were obtained at mRNA expression level (*p* < 0.002) (Figure 1(e)).

**Figure 2:**
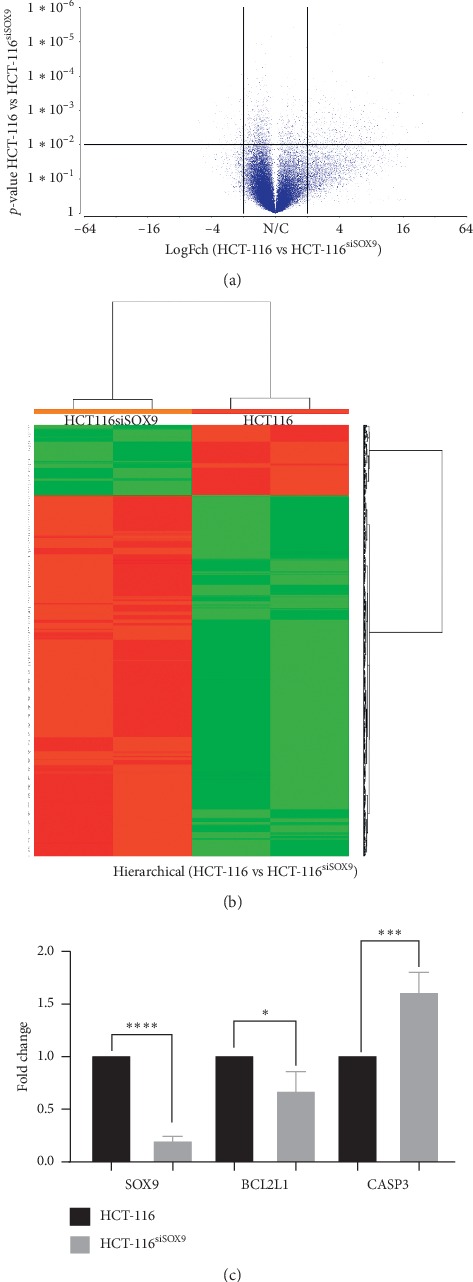
SOX9 silencing deregulates several signaling pathways. Transcriptome profiles of HCT-116 SOX9-silenced and nonsilenced were compared, based on microarray data. (a) In volcano plot, points represent significantly upregulated and downregulated mRNAs in HCT-116siSOX9 with a 2.0-LogFch. (b) Two-dimensional hierarchical clustering of distinguishable mRNAs expression profiles in both groups. Red: higher expression levels; green: lower expression levels. (c) RT-qPCR analysis for microarray data validation confirmed that SOX9 silencing modulates gene expression of selected candidates' genes: SOX9 (^*∗∗∗∗*^*p* < 0.0001), BCL2L1 (^*∗*^*p*=0.37), CASP3 (^*∗∗∗*^*p*=0.0005), and NF1 (^*∗∗*^*p* < 0.0007), in an independent experiment.

To gain insight into the biological functions of SOX9, the gene expression profile of HCT-116^siSOX9^ cells was obtained. Analysis of the original normalized microarrays dataset revealed a total of 369 overexpressed and 151 downregulated genes (LogFch >2 or <–2, adjusted *p* < 0.01) (Figures 2(a) and 2(b)). The full list of deregulated genes is provided in Supplementary Materials (Table S1). Functional analysis reported seven clusters with an enrichment score (ES) greater than 2: nucleosome core (ES 8.71), transcription regulation (ES 7.63), apoptosis regulation (ES 3.23), beta-catenin-TFC complex assembly (ES 2.97), cell cycle (ES 2.8), zing finger (ES 2.46), and DNA repair (ES 2.37) (data not shown). The highest changes in gene expression were in APC (LogFch 19.9) and MYC (LogFch–3.0). Interestingly, 25 histones were downregulated, while transcriptional regulators such as DBF4, ATF2, ATRX, and AFF4 were overexpressed. As expected, pathways with overrepresentation were CRC (Figure S1) and WNT signaling pathways (Figure S2), in which APC was present. This is relevant since it is well known that loss of APC function activates the cascade of events that ultimately lead to malignant transformation [31].

Interestingly, string interactome analysis of Table 1 genes (Figure S3) grouped in apoptosis cluster showed a network associating SOX9 with BCL2L1 through JUN. SOX9 is linked directly to CASP according to text mining, and association of BCL2L1 and CASP3 is established based on experimental results. Moreover, interactome suggests TP53 as a master molecule since it interacts directly with BCL2L1, CASP3, APC, MYC, and JUN. Previously, BCL2L1 and CASP3 have been described as cell survival mediators [32].

### 3.3. BCL2L1 and CASP3 Gene Expression Levels Are Regulated by SOX9 in HCT-116 Cells

For microarrays data validation, we evaluated gene expression of apoptosis regulators in independent HCT-116 transfection assays. We found that after silencing of SOX9 (*p* < 0.0001), BCL2L1 was 25% downregulated (*p*=0.037). Besides, CASP3 enhanced its expression level by 75% (*p*=0.0005) (Figure 2(c)), corroborating microarrays' findings. Interestingly, there are no reports indicating the regulation of prosurvival BCL2L1 and antisurvival CASP3 by SOX9 in CRC.

There is evidence that Bcl-2, a member of BCL2 family, is capable to regulate SOX9 through MEK-ERK1/2, maintaining the phenotype of differentiated chondrocytes [33]. Our results suggest that cell survival could be negatively regulated by SOX9 expression since its downregulation promotes a decrease of BCL2L1 and an increase of CASP3 in HCT-116. Accordingly, downregulation of SOX9 produces an increased apoptosis due to Bcl-xL decrease in human chordoma cell lines [34]. SOX9 downregulation in cholangiocarcinoma enhances expression of apoptosis effectors such as CASP3 and CASP8, suggesting that SOX9 might play an important role in the process of apoptosis [35]. Remarkably, SOX9 modification does not affect caspases activities in other types of cancer such as basal cell and prostate carcinoma [36, 37]. Altogether, these data might suggest that SOX9 could regulate induced cell death by directly affecting both CASP3 and BCL2L1 expression in HCT-116.

### 3.4. SOX9/BCL2L1 Axis Is Present in Poorly Diferenciated CRC Cell Lines

**Figure 3:**
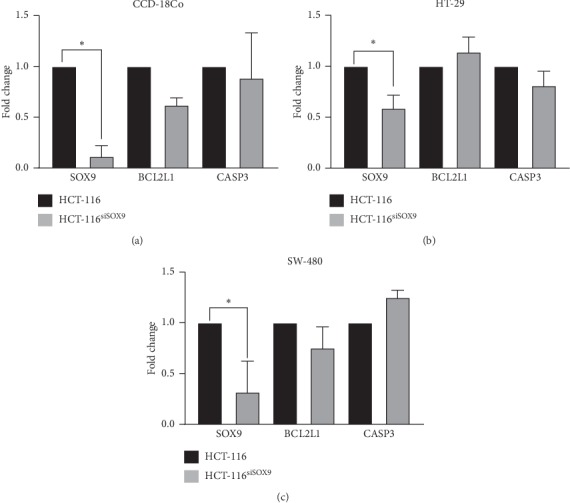
SOX9 regulates BCL2L1 and CASP3 in undifferentiated CRC cell lines. Quantitative RT-qPCR showed that, after SOX9 silencing, BCL2L1 and CASP3 did not change in well-differentiated (a) normal CCD-18Co and (b) HT-29 cell lines, but a tendency similar to HCT-116 was seen in the moderate-differentiated SW-480 (c). However, these changes were not statistically significant. For SOX9 silencing in all cell lines, ^*∗*^*p* < 0.01.

SOX9 regulation of BCL2L1 and CASP3 was evaluated in HT-29 (well-differentiated) and SW-480 (moderate-differentiated) CRC cell lines (Figure 3). We found that, after SOX9 silencing, BCL2L1 and CASP3 did not change in differentiated CCD-18Co and HT-29 (Figures 3(a) and 3(b)), but a similar tendency to that seen in HCT-116 was found in SW-480; however, these changes were not statistically significant (Figure 3(c)).

These data are in agreement with the previous report showing an overexpression of BCL2L1 and downregulation of CASP3 in tumorigenic tissues [38–42]. Poorly differentiated CRCs are more aggressive, does not have an efficient chemotherapy response [43], and shows high proliferation rates and metastasis, having an important impact in survival and prognosis of patients [44].

Our findings demonstrate that SOX9/BCL2L1 axis is present in undifferentiated CRC cell lines, and this could be related to a more aggressive phenotype that resists cell death.

SOX9 downregulation enhances ABT-737-induced apoptosis, but not autophagy in HCT-116.

**Figure 4:**
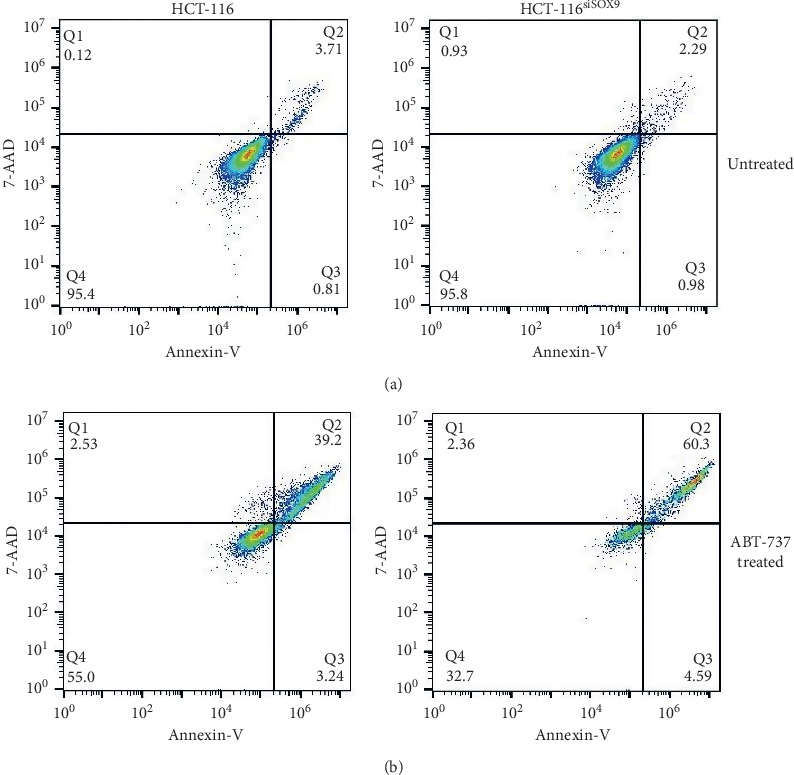
BCL2L1 inhibition increase apoptosis SOX9-silenced HCT-116 cells. Flow cytometry analysis for apoptosis detection through Annexin V and 7-AAD. (a) HCT-116 and HCT-116^siSOX9^ untreated. (b) HCT-116 and HCT-116^siSOX9^ treated with ABT-737.

In order to determine if downregulation of BCL2L1 given by SOX9 silencing produces changes in cell survival, we used ABT-737 as a proapoptotic stimulus. ABT-737 mimics the action of the BH3-only protein BAD by binding the antiapoptotic proteins BCL-2, BCL-xL, and BCL-w and inhibits their function, promoting apoptosis [45]. We found that, in untreated cells, SOX9 silencing has no effect in apoptosis (Figure 4(a)). In cancer conditions, SOX9 and BCL2L1 are overexpressed when compared to normal colon cells (data not shown), and there is a direct relation in their expression since BCL2L1 is a target of SOX9 [46]. However, SOX9 silencing inhibits BCL2L1 expression partially, which can be not enough to induce apoptosis. Besides, there are multiple prosurvival mechanisms [47–50] working at the same time in cancer cells, providing signals that help cells to resist apoptosis. On the contrary, when we compared late apoptosis in ABT-737 treated cells, HCT-116 cells showed 39.2% of apoptosis (*p*=0.0034), and this behavior is enhanced by silencing of SOX9 (60.3%, *p*=0.0003), suggesting a summative inhibition of BCL2L1 expression. These finding suggest that SOX9 plays an important role in cell survival, increasing the expression of prosurvival molecules, such as BCL2L1 in CRC.

Given that BCL2L1 is also an important regulator in autophagy-related mechanisms that promotes cell survival, we evaluated LC3-II protein, a marker of autophagy. In this regard, it has been demonstrated that disruption of BCL2L1-BECN1 complex increases the expression of LC3-II, giving as a result the formation of autophagosomes [51–53].

**Figure 5:**
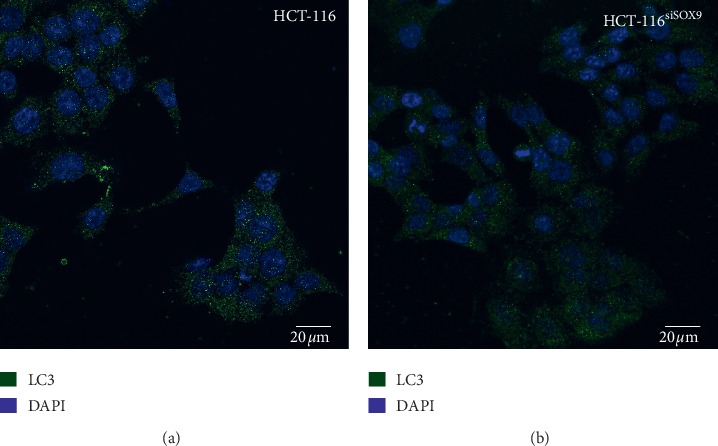
Immunofluorescence assays for autophagy. Autophagosome induction by SOX9/BCL2L1 axis. Micrographies of LC3 protein in HCT-116 and SOX9-silenced HCT-116 cells showed no differences between the groups.

Our results showed that SOX9 silencing does not induce autophagosome formation on HCT-116 cells (Figure 5). Nonetheless, there are multiple mechanisms that are involved in autophagosome formation, involving several molecules such as Ulk1 and 2, Becn1, Atg, LC3, and Tfeb [54–59].

## 4. Conclusions

Differential expression analysis of SOX9-silenced HCT-116 cells showed seven main clusters: nucleosome core, transcription regulation, apoptosis regulation, beta-catenin-TFC complex assembly, cell cycle, zing finger, and DNA repair. APC and MYC showed the highest dysregulation, as well as histones and transcriptional regulators.

SOX9 silencing induced changes in expression patterns of prosurvival BCL2L1 and antisurvival CASP3, suggesting a SOX9 participation in cell survival. Interestingly, a consistent pattern in poorly and moderated-differentiated cell was found.

Finally, inhibition of BCL2L1 is enough to induce apoptosis; however, SOX9 silencing potentiates this effect, but not autophagy, indicating that cell survival could be modulated via SOX9/BCL2L1 axis. To the best of our knowledge, this is the first report suggesting that cell survival in poorly and moderated-differentiated CRC cell lines.

## Figures and Tables

**Table 1:** Differentially expressed apoptosis-related genes.

Gene	Fold change	*p* value
BLID	6.86524	6.81*E* − 05
SOS1	6.34372	0.00854877
TAOK1	6.4378	0.00779361
CASP3	3.37851	0.00672809
CASP8AP2	3.64127	0.00193459
CHUK	3.57394	0.0058005
HIPK3	6.31868	0.00480743
APC	19.9097	0.00037474
BCL2L1	–2.26984	0.00601934
CHAC1	–5.85796	0.00664003
E2F1	–3.63746	0.00398582
JUN	–2.25396	0.0092463
APEX1	–2.28963	0.00293408
BEX2	–2.12243	0.00385886
TP53	–3.04873	0.00540116
MYC	–3.00718	0.00249873
PDPK1	–2.05947	0.00413932

## Data Availability

All data included in this work are available within the manuscript and supplementary materials.

## Supplementary Materials

Supplementary Materials Figure S1: pathway analysis. Pathway with overrepresentation is the CRC pathway in which APC appears. Interestingly, APC expression is restored when SOX9 is silenced according to DE analysis. Figure S2: pathway analysis. Pathway with overrepresentation is the WNT signaling pathway in which APC appears. Interestingly, APC expression is restored when SOX9 is silenced according to DE analysis. Figure S3: string interactome (https://string-db.org/cgi/network.pl?taskId=Y8RKNUbzndpT). In Table 1, genes grouped in apoptosis cluster shows a network associating SOX9 with BCL2L1 through JUN. Besides, SOX9 is linked directly to CASP according to text mining and association of BCL2L1 and CASP3 is established based on experimental results. Table S1: differentially expressed coding genes between HCT-116 vs. HCT-116siSOX9 selected based on a fold-change of 2 in absolute value, the genes with an adjusted *p* value <0.01.
