# Supplementary material for: Cell Survival Is Regulated via SOX9/BCL2L1 Axis in HCT-116 Colorectal Cancer Cell Line
Source: J Oncol. 2020 Apr 29;2020:5701527. doi: 10.1155/2020/5701527 (PMC7206885; doi:10.1155/2020/5701527)
Supplement: Supplementary Materials — Figure S1: pathway analysis. Pathway with overrepresentation is the CRC pathway in which APC appears. Interestingly, APC expression is restored when SOX9 is silenced according to DE analysis. Figure S2: pathway analysis. Pathway with overrepresentation is the WNT signaling pathway in which APC appears. Interestingly, APC expression is restored when SOX9 is silenced according to DE analysis. Figure S3: string interactome (https://string-db.org/cgi/network.pl?taskId=Y8RKNUbzndpT). In Table 1, genes grouped in apoptosis cluster shows a network associating SOX9 with BCL2L1 through JUN. Besides, SOX9 is linked directly to CASP according to text mining and association of BCL2L1 and CASP3 is established based on experimental results. Table S1: differentially expressed coding genes between HCT-116 vs. HCT-116siSOX9 selected based on a fold-change of 2 in absolute value, the genes with an adjusted p value <0.01. [file 5701527.f1.docx]

### Supplementary Figures


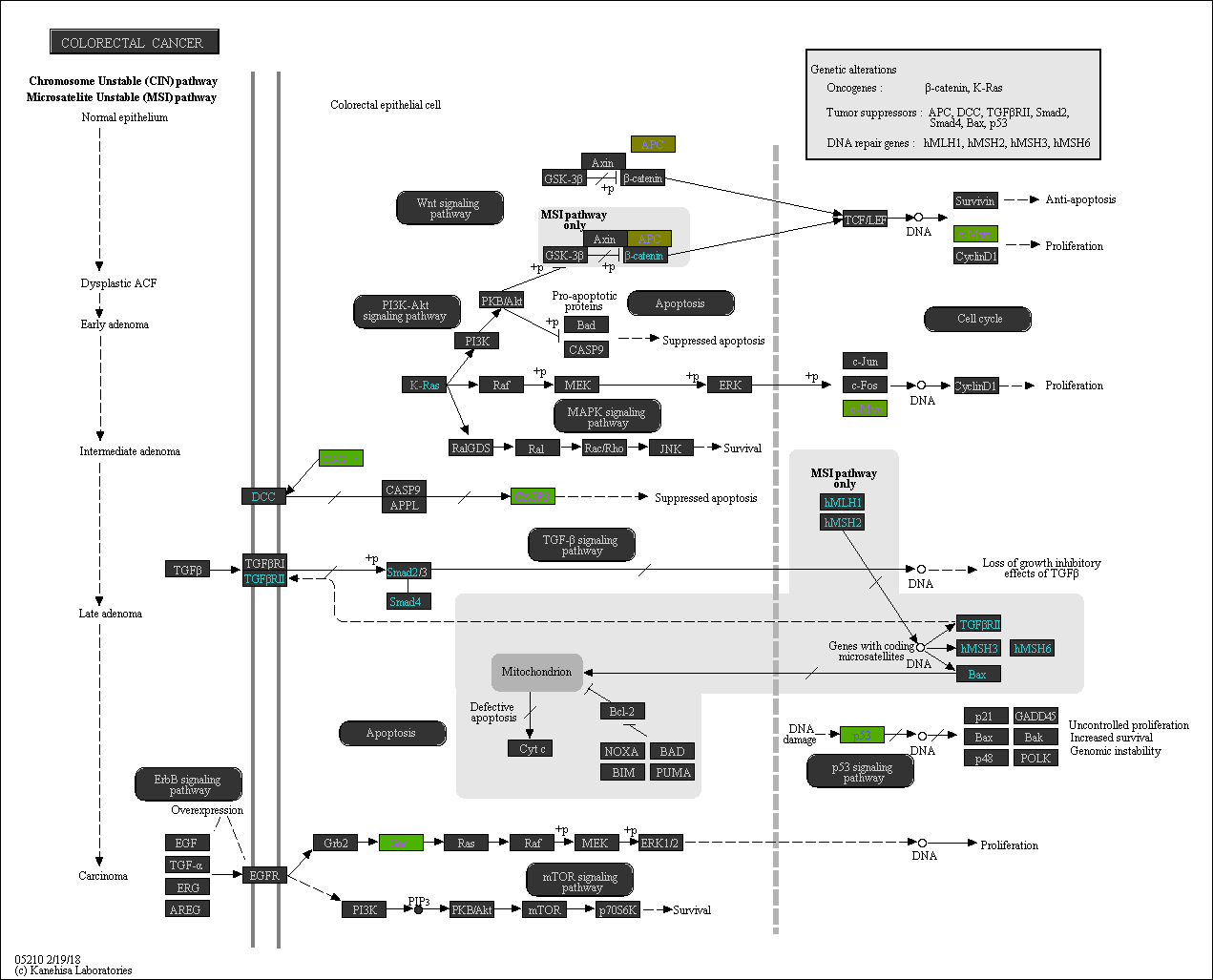


**Figure S1. Pathway analysis.** Pathways with overrepresentation is CRC pathway in which APC appears. Interestingly, APC expression is restored when SOX9 is silenced according to DE analysis.

**
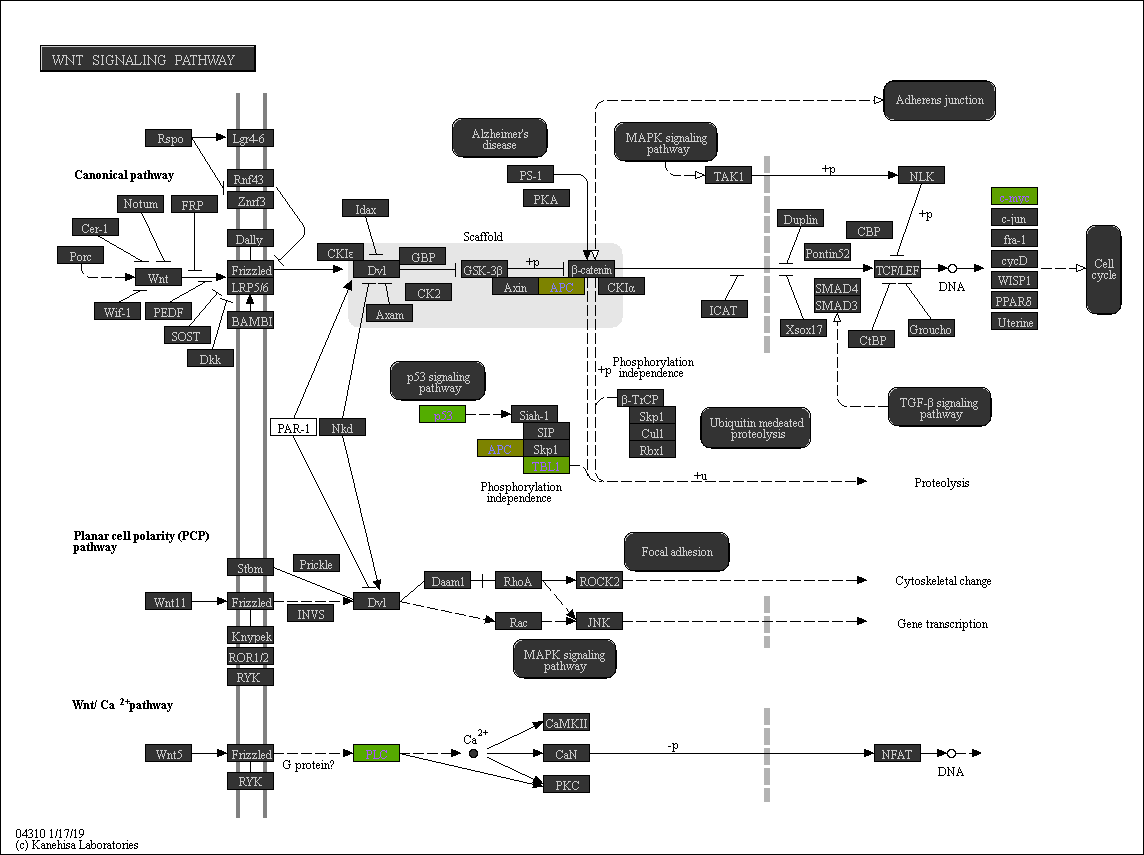
**

**Figure S2**. **Pathway analysis.** Pathways with overrepresentation is WNT signaling pathway in which APC appears. Interestingly, APC expression is restored when SOX9 is silenced according to DE analysis.

**Figure S3. String Interactome.** <https://string-db.org/cgi/network.pl?taskId=Y8RKNUbzndpT>. Table 1 genes, grouped in Apoptosis cluster, shows a network associating SOX9 with BCL2L1 through JUN. Besides, SOX9 is linked directly to CASP according to text mining and association of BCL2L1 and CASP3 is stablished based on experimental results.

### Supplementary Table

**Table S1.** Differentially expressed coding genes between HCT-116 vs HCT-116^siSOX9^ selected based on a fold-change of 2 in absolute value, the genes with an adjusted p-value < 0.01

| Gene | ID | Accession Number | Fold-change | P-value | Expression |
| --- | --- | --- | --- | --- | --- |
| ZBTB41 | TC0100016833.hg.1 | NM_194314 | 27,7969 | 0,00788955 | INCREASED |
| MANEA | TC0600008863.hg.1 | NM_024641 | 20,9843 | 0,00106213 | INCREASED |
| APC | TC0500013211.hg.1 | NM_000038 | 19,9097 | 0,00037474 | INCREASED |
| PHIP | TC0600012358.hg.1 | NM_017934 | 18,152 | 0,00074037 | INCREASED |
| RB1CC1 | TC0800010427.hg.1 | NM_001083617 | 16,7129 | 0,00090563 | INCREASED |
| ANKRD50 | TC0400011791.hg.1 | NM_001167882 | 16,5689 | 0,00488108 | INCREASED |
| CCDC186 | TC1000011892.hg.1 | NM_018017 | 16,1185 | 0,00678458 | INCREASED |
| KIAA1033 | TC1200008706.hg.1 | NM_001293640 | 15,7732 | 0,00075129 | INCREASED |
| IL6ST | TC0500010788.hg.1 | NM_001190981 | 15,5639 | 0,00061057 | INCREASED |
| CENPF | TC0100011581.hg.1 | NM_016343 | 15,2311 | 0,00778116 | INCREASED |
| UACA | TC1500009921.hg.1 | NM_001008224 | 15,1655 | 0,00305191 | INCREASED |
| VPS13A | TC0900007646.hg.1 | NM_001018037 | 14,9023 | 0,00031543 | INCREASED |
| SAMD9 | TC0700011796.hg.1 | NM_001193307 | 14,8146 | 0,00917327 | INCREASED |
| FAM171B | TC0200010219.hg.1 | NM_177454 | 14,1365 | 0,00115597 | INCREASED |
| DST | TC0600012123.hg.1 | NM_001144769 | 13,6716 | 0,00630102 | INCREASED |
| LYSMD3 | TC0500011435.hg.1 | NM_001286812 | 13,1017 | 0,00975753 | INCREASED |
| CCDC88A | TC0200012643.hg.1 | NM_001135597 | 12,763 | 0,0091997 | INCREASED |
| CWC22 | TC0200015099.hg.1 | NM_020943 | 12,7193 | 0,00470262 | INCREASED |
| TTC37 | TC0500011490.hg.1 | NM_014639 | 12,4987 | 0,00026242 | INCREASED |
| EEA1 | TC1200011519.hg.1 | NM_003566 | 12,4653 | 2,92E-05 | INCREASED |
| VPS13C | TC1500009669.hg.1 | NM_001018088 | 12,0833 | 0,00075405 | INCREASED |
| GCC2 | TC0200008803.hg.1 | NM_181453 | 12,064 | 0,00044453 | INCREASED |
| TRIP11 | TC1400010778.hg.1 | NM_004239 | 12,0025 | 0,0033934 | INCREASED |
| THOC2 | TC0X00010712.hg.1 | NM_001081550 | 11,7736 | 0,00647832 | INCREASED |
| CAMSAP2 | TC0100011148.hg.1 | NM_001297707 | 11,7612 | 0,0028685 | INCREASED |
| SCYL2 | TC1200008592.hg.1 | NM_017988 | 11,7394 | 0,00535545 | INCREASED |
| STXBP4 | TC1700008328.hg.1 | NM_178509 | 11,6812 | 0,0014523 | INCREASED |
| CEP350 | TC0100010793.hg.1 | NM_014810 | 11,6039 | 0,00173455 | INCREASED |
| PHF3 | TC0600014143.hg.1 | NM_001290259 | 11,5607 | 0,00593076 | INCREASED |
| FAR1 | TC1100006906.hg.1 | NM_032228 | 11,4639 | 0,00664853 | INCREASED |
| ATRX | TC0X00010126.hg.1 | NM_000489 | 11,4328 | 0,00751444 | INCREASED |
| AHI1 | TC0600013258.hg.1 | NM_001134830 | 11,4302 | 0,00057638 | INCREASED |
| UTRN | TC0600009712.hg.1 | NM_007124 | 11,3113 | 0,00783572 | INCREASED |
| PUS7L | TC1200012787.hg.1 | NM_001098614 | 10,7499 | 0,0055688 | INCREASED |
| TMTC3 | TC1200008373.hg.1 | NM_181783 | 10,5426 | 0,00829471 | INCREASED |
| KTN1 | TC1400007244.hg.1 | NM_001079521 | 10,5333 | 0,00439373 | INCREASED |
| SMC5 | TC0900007520.hg.1 | NM_015110 | 10,4315 | 0,00920817 | INCREASED |
| EXOC5 | TC1400009295.hg.1 | NM_006544 | 10,262 | 0,00536988 | INCREASED |
| ZNF518A | TC1000008535.hg.1 | NM_001278524 | 10,2591 | 0,00432654 | INCREASED |
| CLOCK | TC0400010745.hg.1 | NM_001267843 | 10,2453 | 0,00926924 | INCREASED |
| FER | TC0500008278.hg.1 | NM_001308028 | 10,2333 | 0,00085633 | INCREASED |
| PTAR1 | TC0900010352.hg.1 | NM_001099666 | 10,0774 | 0,00620887 | INCREASED |
| SENP7 | TC0300011853.hg.1 | NM_001077203 | 9,87934 | 0,00146661 | INCREASED |
| MOSPD2 | TC0X00006671.hg.1 | NM_001177475 | 9,86388 | 0,00184857 | INCREASED |
| OR51K1P | TC1100009928.hg.1 | OTTHUMT00000143353 | 9,70819 | 0,00448641 | INCREASED |
| GOLGB1 | TC0300012170.hg.1 | NM_001256486 | 9,61954 | 0,00693797 | INCREASED |
| SASS6 | TC0100015058.hg.1 | NM_001304829 | 9,26495 | 0,00440046 | INCREASED |
| DZIP3 | TC0300008289.hg.1 | NM_014648 | 8,7839 | 0,00636018 | INCREASED |
| USP47 | TC1100006860.hg.1 | NM_001282659 | 8,67151 | 0,00460097 | INCREASED |
| TET2 | TC0400008318.hg.1 | NM_001127208 | 8,66351 | 0,00011921 | INCREASED |
| GPR180 | TC1300007731.hg.1 | NM_180989 | 8,53806 | 0,00829936 | INCREASED |
| UFL1 | TC0600008873.hg.1 | NM_015323 | 8,51415 | 0,00602843 | INCREASED |
| TBC1D32 | TC0600013033.hg.1 | NM_152730 | 8,41236 | 0,0005273 | INCREASED |
| LIFR | TC0500010540.hg.1 | NM_001127671 | 8,39818 | 0,00775413 | INCREASED |
| ZBTB38 | TC0300008999.hg.1 | NM_001080412 | 8,36942 | 0,00722604 | INCREASED |
| BAZ2B | TC0200014719.hg.1 | NM_001289975 | 8,1852 | 0,00334666 | INCREASED |
| OR51I1 | TC1100009929.hg.1 | NM_001005288 | 8,12108 | 0,00439507 | INCREASED |
| KIAA1551 | TC1200007251.hg.1 | NM_018169 | 8,099 | 0,002352 | INCREASED |
| OR51B3P | TC1100009925.hg.1 | OTTHUMT00000142984 | 8,08674 | 0,00435057 | INCREASED |
| NRIP1 | TC2100008545.hg.1 | NM_003489 | 8,06176 | 0,00064822 | INCREASED |
| VPS54 | TC0200012804.hg.1 | NM_001005739 | 8,05383 | 0,00098655 | INCREASED |
| CAPS2 | TC1200011298.hg.1 | NM_001286547 | 7,86525 | 0,00112351 | INCREASED |
| CEP170 | TC0100017974.hg.1 | NM_001042404 | 7,83934 | 0,00380821 | INCREASED |
| KIF18A | TC1100010418.hg.1 | NM_031217 | 7,74246 | 0,00063414 | INCREASED |
| CWC27 | TC0500007591.hg.1 | NM_001297644 | 7,7207 | 0,0019356 | INCREASED |
| KIF16B | TC2000008473.hg.1 | NM_001199865 | 7,7187 | 0,00357535 | INCREASED |
| AHCTF1P1 | TC0200014758.hg.1 | NR_077058 | 7,66163 | 0,00350724 | INCREASED |
| ZFYVE16 | TC0500007912.hg.1 | NM_001105251 | 7,55317 | 0,00165992 | INCREASED |
| SOCS4 | TC1400007221.hg.1 | NM_080867 | 7,53732 | 0,00586842 | INCREASED |
| NAA15 | TC0400012846.hg.1 | NM_057175 | 7,52613 | 0,0082482 | INCREASED |
| SHOC2 | TC1000008908.hg.1 | NM_001269039 | 7,5216 | 0,00880563 | INCREASED |
| ESCO1 | TC1800008231.hg.1 | NM_052911 | 7,50067 | 0,00069585 | INCREASED |
| SUCO | TC0100010623.hg.1 | NM_001282750 | 7,45706 | 0,00175027 | INCREASED |
| BBX | TC0300008271.hg.1 | NM_001142568 | 7,44218 | 0,00618262 | INCREASED |
| SEP07 | TC0700007178.hg.1 | NM_001011553 | 7,42457 | 0,00567162 | INCREASED |
| SEC62 | TC0300009458.hg.1 | NM_003262 | 7,41096 | 0,0037912 | INCREASED |
| DDX60 | TC0400012366.hg.1 | NM_017631 | 7,40665 | 0,00670991 | INCREASED |
| PPIG | TC0200009911.hg.1 | NM_004792 | 7,30633 | 0,0027458 | INCREASED |
| STAG2 | TC0X00008332.hg.1 | NM_001042749 | 7,2654 | 0,00624901 | INCREASED |
| CEP290 | TC1200011454.hg.1 | NM_025114 | 7,25907 | 0,00725673 | INCREASED |
| ARID5B | TC1000007761.hg.1 | NM_001244638 | 7,25695 | 0,00605166 | INCREASED |
| RABGAP1L | TC0100010672.hg.1 | NM_001035230 | 7,20063 | 0,00620396 | INCREASED |
| ERV3-1 | TC0700011318.hg.1 | NM_001007253 | 7,15764 | 0,00869616 | INCREASED |
| SESTD1 | TC0200015087.hg.1 | NM_178123 | 7,14274 | 0,00609064 | INCREASED |
| CWF19L2 | TC1100012208.hg.1 | NM_152434 | 7,14078 | 0,00204658 | INCREASED |
| ZEB1 | TC1000007199.hg.1 | NM_001128128 | 7,10707 | 0,00544853 | INCREASED |
| DDX60L | TC0400012991.hg.1 | ENST00000512958 | 7,052 | 0,00940708 | INCREASED |
| DCUN1D1 | TC0300013330.hg.1 | NM_001308101 | 7,05096 | 0,00300326 | INCREASED |
| FAM63B | TC1500007379.hg.1 | NM_001040450 | 6,9764 | 0,00641846 | INCREASED |
| KITLG | TC1200011460.hg.1 | NM_000899 | 6,96525 | 0,00406152 | INCREASED |
| ELMOD2 | TC0400008841.hg.1 | NM_153702 | 6,92221 | 0,00070893 | INCREASED |
| VAMP7 | TC0X00008884.hg.1 | NM_001145149 | 6,90672 | 0,00298289 | INCREASED |
| ZFC3H1 | TC1200011255.hg.1 | NM_144982 | 6,87607 | 0,00637969 | INCREASED |
| BLID | TC1100012583.hg.1 | NM_001001786 | 6,86524 | 6,81E-05 | INCREASED |
| ARID4A | TC1400007307.hg.1 | NM_002892 | 6,84065 | 0,00844882 | INCREASED |
| FGD6 | TC1200011574.hg.1 | NM_018351 | 6,83963 | 0,00027364 | INCREASED |
| ZDHHC2 | TC0800006864.hg.1 | NM_016353 | 6,82592 | 0,00384273 | INCREASED |
| METTL15 | TC1100007139.hg.1 | NM_001113528 | 6,80464 | 0,00954875 | INCREASED |
| TMF1 | TC0300011450.hg.1 | NM_007114 | 6,74851 | 0,00738142 | INCREASED |
| RAB11FIP2 | TC1000011964.hg.1 | NM_014904 | 6,73599 | 0,00311846 | INCREASED |
| RSF1 | TC1100011723.hg.1 | NM_016578 | 6,73393 | 0,00163694 | INCREASED |
| FOXN2 | TC0200007533.hg.1 | NM_002158 | 6,70575 | 0,00098717 | INCREASED |
| SEMA3C | TC0700011626.hg.1 | NM_006379 | 6,65482 | 0,00427627 | INCREASED |
| ZBTB20 | TC0300012048.hg.1 | NM_001164342 | 6,58762 | 0,00414225 | INCREASED |
| SCFD1 | TC1400006823.hg.1 | NM_001257376 | 6,55461 | 0,00903907 | INCREASED |
| CTD-2014D20.1 | TC0800012010.hg.1 | ENST00000623208 | 6,54365 | 0,00371966 | INCREASED |
| PRKAA2 | TC0100008450.hg.1 | NM_006252 | 6,53473 | 0,00808142 | INCREASED |
| VAMP7 | TC0Y00006858.hg.1 | NM_001145149_2 | 6,52928 | 0,00696857 | INCREASED |
| POLR3G | TC0500008054.hg.1 | NM_006467 | 6,52623 | 0,00131742 | INCREASED |
| TAOK1 | TC1700007419.hg.1 | NM_020791 | 6,4378 | 0,00779361 | INCREASED |
| SCOC | TC0400008834.hg.1 | NM_001153446 | 6,38872 | 0,00726363 | INCREASED |
| SOS1 | TC0200012307.hg.1 | NM_005633 | 6,34372 | 0,00854877 | INCREASED |
| TAS2R30 | TC1200012763.hg.1 | NM_001097643 | 6,33313 | 0,00495584 | INCREASED |
| HIPK3 | TC1100007227.hg.1 | NM_001048200 | 6,31868 | 0,00480743 | INCREASED |
| FAM83B | TC0600008319.hg.1 | NM_001010872 | 6,31779 | 0,00377739 | INCREASED |
| COPS2 | TC1500009363.hg.1 | NM_001143887 | 6,27854 | 0,00547838 | INCREASED |
| ZNF567 | TC1900007950.hg.1 | NM_001300979 | 6,27165 | 0,00587908 | INCREASED |
| NIPBL | TC0500007163.hg.1 | NM_015384 | 6,24127 | 0,00016735 | INCREASED |
| LARP7 | TC0400008443.hg.1 | NM_001267039 | 6,04654 | 0,00349905 | INCREASED |
| PYROXD1 | TC1200007051.hg.1 | NM_024854 | 6,03925 | 0,00286347 | INCREASED |
| FAT4 | TC0400008641.hg.1 | NM_001291285 | 5,98846 | 0,00912504 | INCREASED |
| PANK3 | TC0500013399.hg.1 | NM_024594 | 5,9688 | 0,00761202 | INCREASED |
| PRPF40A | TC0200014631.hg.1 | NM_017892 | 5,96811 | 0,00683386 | INCREASED |
| LCORL | TC0400010168.hg.1 | NM_001166139 | 5,95745 | 0,00213029 | INCREASED |
| KIF20B | TC1000008406.hg.1 | NM_001284259 | 5,90027 | 0,00105453 | INCREASED |
| N4BP2 | TC0400007306.hg.1 | NM_018177 | 5,786 | 0,00292106 | INCREASED |
| ZNF510 | TC0900010922.hg.1 | NM_014930 | 5,67433 | 0,00101526 | INCREASED |
| MTERF1 | TC0700013583.hg.1 | NM_001301134 | 5,65546 | 0,00052377 | INCREASED |
| MOB1B | TC0400012810.hg.1 | NM_001244766 | 5,59798 | 0,00200233 | INCREASED |
| ARHGAP42 | TC1100008881.hg.1 | NM_152432 | 5,56081 | 0,00472878 | INCREASED |
| SNX16 | TC0800010949.hg.1 | NM_022133 | 5,53918 | 0,00081916 | INCREASED |
| DDHD1 | TC1400009198.hg.1 | NM_001160147 | 5,52756 | 0,00992796 | INCREASED |
| ZC2HC1A | TC0800008032.hg.1 | NM_016010 | 5,5169 | 0,00462604 | INCREASED |
| KDM7A | TC0700012798.hg.1 | NM_030647 | 5,4712 | 0,00194357 | INCREASED |
| PHACTR2 | TC0600014188.hg.1 | NM_001100164 | 5,42295 | 0,00265415 | INCREASED |
| ZNF138 | TC0700013371.hg.1 | NM_001160183 | 5,39752 | 0,00099946 | INCREASED |
| GDAP2 | TC0100015434.hg.1 | NM_001135589 | 5,39262 | 0,00269337 | INCREASED |
| THUMPD1 | TC1600009666.hg.1 | NM_001304550 | 5,3765 | 0,00846343 | INCREASED |
| PGAP1 | TC0200015332.hg.1 | NM_024989 | 5,36944 | 0,00897727 | INCREASED |
| MOB4 | TC0200016585.hg.1 | NM_001100819 | 5,35638 | 0,00936125 | INCREASED |
| LACC1 | TC1300009956.hg.1 | NM_001128303 | 5,33808 | 0,00793588 | INCREASED |
| MYNN | TC0300009451.hg.1 | NM_001185118 | 5,3374 | 0,00984807 | INCREASED |
| DENND1B | TC0100016839.hg.1 | NM_001195215 | 5,32171 | 0,00026067 | INCREASED |
| KLF12 | TC1300009249.hg.1 | NM_007249 | 5,26107 | 0,00088845 | INCREASED |
| CRYBG3 | TC0300008088.hg.1 | NM_153605 | 5,23979 | 0,00437808 | INCREASED |
| ICE2 | TC1500009639.hg.1 | NM_001018089 | 5,21677 | 0,00092216 | INCREASED |
| ANKRD36B | TC0200013567.hg.1 | NM_025190 | 5,17962 | 0,00647078 | INCREASED |
| CUL4B | TC0X00010675.hg.1 | NM_001079872 | 5,16236 | 0,00410272 | INCREASED |
| PSMD6-AS2 | TC0300007681.hg.1 | NR_038286 | 5,15343 | 0,00165054 | INCREASED |
| ZNF107 | TC0700013370.hg.1 | NM_001013746 | 5,14329 | 0,003719 | INCREASED |
| BNIP3P7 | TC1300006438.hg.1 | ENST00000605641 | 5,1281 | 0,00214683 | INCREASED |
| PLS1 | TC0300009033.hg.1 | NM_001145319 | 5,11301 | 0,00277556 | INCREASED |
| CUL5 | TC1100008979.hg.1 | NM_003478 | 5,10841 | 0,00670011 | INCREASED |
| MIA3 | TC0100011692.hg.1 | NM_001300867 | 5,10567 | 0,00601536 | INCREASED |
| SNX4 | TC0300012252.hg.1 | NM_003794 | 5,0943 | 0,00054517 | INCREASED |
| ARAP2 | TC0400012917.hg.1 | NM_015230 | 5,08552 | 0,00869289 | INCREASED |
| STXBP5 | TC0600009753.hg.1 | NM_001127715 | 5,019 | 0,00499925 | INCREASED |
| EDEM3 | TC0100016676.hg.1 | NM_025191 | 4,9999 | 0,00973083 | INCREASED |
| GPR52 | TC0100010674.hg.1 | NM_005684 | 4,99215 | 0,00126546 | INCREASED |
| TANK | TC0200009806.hg.1 | NM_001199135 | 4,98479 | 0,00502252 | INCREASED |
| SACS | TC1300008329.hg.1 | NM_001278055 | 4,90428 | 0,00127211 | INCREASED |
| TNPO1P1 | TC1000008278.hg.1 | OTTHUMT00000049130 | 4,8358 | 0,00436754 | INCREASED |
| NHLRC2 | TC1000008961.hg.1 | NM_198514 | 4,80033 | 0,00831145 | INCREASED |
| PIKFYVE | TC0200010624.hg.1 | NM_001178000 | 4,79092 | 0,00993279 | INCREASED |
| ZNF148 | TC0300012245.hg.1 | NM_021964 | 4,78733 | 0,00035221 | INCREASED |
| ACADSB | TC1000009169.hg.1 | NM_001609 | 4,69774 | 0,00799639 | INCREASED |
| USP8 | TC1500007202.hg.1 | NM_001128610 | 4,69364 | 0,00735601 | INCREASED |
| PRR14L | TC2200008489.hg.1 | NM_173566 | 4,68683 | 0,00851287 | INCREASED |
| PPP4R3B | TC0200012648.hg.1 | NM_001122964 | 4,68197 | 0,00014396 | INCREASED |
| MED13 | TC1700011369.hg.1 | NM_005121 | 4,67852 | 0,00968552 | INCREASED |
| CTAGE9 | TC0600013180.hg.1 | NM_001145659 | 4,66655 | 0,00577649 | INCREASED |
| NAMPTP3 | TC1600007657.hg.1 | ENST00000569273 | 4,63398 | 0,00959726 | INCREASED |
| XRCC4 | TC0500007966.hg.1 | NM_003401 | 4,60048 | 0,00343875 | INCREASED |
| CDC73 | TC0100011027.hg.1 | NM_024529 | 4,60033 | 0,00157019 | INCREASED |
| ZC3H13 | TC1300008837.hg.1 | NM_015070 | 4,58817 | 0,00082622 | INCREASED |
| NBN | TC0800011061.hg.1 | NM_001024688 | 4,58686 | 0,00854629 | INCREASED |
| DBF4 | TC0700008263.hg.1 | NM_006716 | 4,58683 | 0,00869976 | INCREASED |
| TRIM23 | TC0500010930.hg.1 | NM_001656 | 4,54068 | 0,00615864 | INCREASED |
| LMLN | TC0300010077.hg.1 | NM_001136049 | 4,50056 | 0,00924807 | INCREASED |
| ANKRD26 | TC1000010113.hg.1 | NM_001256053 | 4,46035 | 0,00517091 | INCREASED |
| TAS2R10 | TC1200009919.hg.1 | NM_023921 | 4,46005 | 0,00563982 | INCREASED |
| MYH15 | TC0300011941.hg.1 | NM_014981 | 4,45459 | 0,00155517 | INCREASED |
| LIG4 | TC1300009740.hg.1 | NM_001098268 | 4,44072 | 0,00994591 | INCREASED |
| CCDC68 | TC1800008743.hg.1 | NM_001143829 | 4,37217 | 0,00836903 | INCREASED |
| TTC21B | TC0200016747.hg.1 | NM_024753 | 4,3378 | 0,00618006 | INCREASED |
| NEK1 | TC0400012381.hg.1 | NM_001199397 | 4,30152 | 0,00559271 | INCREASED |
| CTAGE8 | TC0700012911.hg.1 | ENST00000487179 | 4,2985 | 0,00901488 | INCREASED |
| PTBP2 | TC0100009142.hg.1 | NM_001300985 | 4,29663 | 0,00989519 | INCREASED |
| B3GNT5 | TC0300009661.hg.1 | NM_032047 | 4,29592 | 0,00312857 | INCREASED |
| COL4A3BP | TC0500011163.hg.1 | NM_001130105 | 4,27736 | 0,00252816 | INCREASED |
| FEM1C | TC0500011751.hg.1 | NM_020177 | 4,2686 | 0,00475171 | INCREASED |
| COMMD8 | TC0400010606.hg.1 | NM_017845 | 4,26409 | 0,00999199 | INCREASED |
| ATF2 | TC0200015009.hg.1 | NM_001256090 | 4,26399 | 0,0026259 | INCREASED |
| ARL5A | TC0200016740.hg.1 | NM_001037174 | 4,25781 | 0,00999132 | INCREASED |
| GABPA | TC2100006787.hg.1 | NM_001197297 | 4,22188 | 0,00136595 | INCREASED |
| RTTN | TC1800008978.hg.1 | NM_173630 | 4,21545 | 0,00993759 | INCREASED |
| ZNF780A | TC1900011952.hg.1 | NM_001010880 | 4,20811 | 0,0068141 | INCREASED |
| ZNF449 | TC0X00008509.hg.1 | NM_152695 | 4,19836 | 0,00111338 | INCREASED |
| SLC44A5 | TC0100014617.hg.1 | NM_001130058 | 4,18764 | 0,00883772 | INCREASED |
| MTCYBP28 | TC1600011005.hg.1 | ENST00000566728 | 4,16284 | 0,00650928 | INCREASED |
| ITSN2 | TC0200011980.hg.1 | NM_006277 | 4,15132 | 0,00833813 | INCREASED |
| BEND6 | TC0600008343.hg.1 | NM_152731 | 4,14962 | 0,0001915 | INCREASED |
| IFNE | TC0900009711.hg.1 | NM_176891 | 4,12923 | 0,00613728 | INCREASED |
| RBM46 | TC0400009074.hg.1 | NM_001277171 | 4,11241 | 0,00220042 | INCREASED |
| ADAM10 | TC1500009574.hg.1 | NM_001110 | 4,1093 | 0,00043263 | INCREASED |
| REV3L | TC0600012863.hg.1 | NM_001286431 | 4,09963 | 0,00518451 | INCREASED |
| SRFBP1 | TC0500008467.hg.1 | NM_152546 | 4,07178 | 0,00410086 | INCREASED |
| ZNF254 | TC1900007622.hg.1 | NM_001278661 | 4,06002 | 0,00313793 | INCREASED |
| ATP5A1P10 | TC0900007450.hg.1 | ENST00000622403 | 4,0029 | 3,05E-06 | INCREASED |
| ZNF518B | TC0400010048.hg.1 | NM_053042 | 4,00185 | 0,00713217 | INCREASED |
| RAB30 | TC1100011797.hg.1 | NM_001286059 | 3,99104 | 0,00407068 | INCREASED |
| STARD4 | TC0500011702.hg.1 | NM_001308056 | 3,98541 | 0,00158023 | INCREASED |
| TMEM65 | TC0800012457.hg.1 | NM_194291 | 3,95915 | 0,00095896 | INCREASED |
| C1GALT1 | TC0700006662.hg.1 | NM_020156 | 3,95413 | 0,00060848 | INCREASED |
| NSRP1 | TC1700007444.hg.1 | NM_001261467 | 3,91584 | 0,00447778 | INCREASED |
| EBAG9P1 | TC1000011591.hg.1 | ENST00000418955 | 3,89103 | 0,00351991 | INCREASED |
| MTATP6P19 | TC0X00007984.hg.1 | ENST00000413797 | 3,88065 | 0,00781564 | INCREASED |
| HIBCH | TC0200015225.hg.1 | NM_014362 | 3,81298 | 0,00899942 | INCREASED |
| OR5AW1P | TC0X00010820.hg.1 | ENST00000426334 | 3,80891 | 0,00876655 | INCREASED |
| WDR63 | TC0100008897.hg.1 | NM_001288563 | 3,78812 | 0,00279333 | INCREASED |
| GPR155 | TC0200014991.hg.1 | NM_001033045 | 3,78633 | 0,00271632 | INCREASED |
| DNAJB14 | TC0400011422.hg.1 | NM_001031723 | 3,78141 | 0,0033176 | INCREASED |
| GOLGA5 | TC1400008019.hg.1 | NM_005113 | 3,76314 | 0,00721704 | INCREASED |
| RBM41 | TC0X00010462.hg.1 | NM_001171080 | 3,756 | 0,00435449 | INCREASED |
| REL | TC0200016452.hg.1 | NM_001291746 | 3,74939 | 0,00382731 | INCREASED |
| USP15 | TC1200007954.hg.1 | NM_001252078 | 3,74782 | 0,00179116 | INCREASED |
| ZBTB44 | TC1100012831.hg.1 | NM_001301098 | 3,74444 | 0,00214707 | INCREASED |
| WBP5 | TC0X00008001.hg.1 | NM_001006612 | 3,72449 | 0,00349292 | INCREASED |
| ELF1 | TC1300008708.hg.1 | hsa_circ_0000477 | 3,71449 | 0,00947913 | INCREASED |
| ARL6 | TC0300008086.hg.1 | NM_001278293 | 3,71394 | 0,00235196 | INCREASED |
| TET1 | TC1000007861.hg.1 | NM_030625 | 3,70717 | 0,00868718 | INCREASED |
| FAM214A | TC1500009460.hg.1 | NM_001286495 | 3,69913 | 0,00486887 | INCREASED |
| KIAA0895 | TC0700010749.hg.1 | NM_001100425 | 3,67712 | 0,00124916 | INCREASED |
| IFT88 | TC1300006503.hg.1 | NM_006531 | 3,65264 | 0,00925884 | INCREASED |
| CASP8AP2 | TC0600008780.hg.1 | NM_001137667 | 3,64127 | 0,00193459 | INCREASED |
| OFD1 | TC0X00006658.hg.1 | NM_003611 | 3,62452 | 0,00221418 | INCREASED |
| RPL12P24 | TC0800011415.hg.1 | ENST00000474907 | 3,62234 | 0,00372672 | INCREASED |
| MRPL1 | TC0400007923.hg.1 | NM_020236 | 3,59993 | 0,00630895 | INCREASED |
| ZC3H6 | TC0200008911.hg.1 | NM_198581 | 3,59961 | 5,90E-06 | INCREASED |
| CHUK | TC1000012585.hg.1 | NM_001278 | 3,57394 | 0,0058005 | INCREASED |
| MID2 | TC0X00008078.hg.1 | NM_012216 | 3,56328 | 0,00703502 | INCREASED |
| RC3H2 | TC0900012272.hg.1 | NM_001100588 | 3,55469 | 0,00644831 | INCREASED |
| ZNF616 | TC1900012022.hg.1 | NM_178523 | 3,55218 | 0,00222 | INCREASED |
| ZNF101P2 | TC0100016784.hg.1 | ENST00000439152 | 3,52589 | 0,0008553 | INCREASED |
| DNM3 | TC0100010609.hg.1 | NM_001136127 | 3,50176 | 0,00357848 | INCREASED |
| PTPN4 | TC0200009074.hg.1 | NM_002830 | 3,49935 | 0,00414958 | INCREASED |
| SEC24A | TC0500008700.hg.1 | NM_001252231 | 3,47629 | 0,00098037 | INCREASED |
| TBL1XR1 | TC0300013215.hg.1 | NM_024665 | 3,47177 | 0,00213275 | INCREASED |
| EHBP1 | TC0200007759.hg.1 | NM_001142614 | 3,44481 | 0,0074421 | INCREASED |
| GAB1 | TC0400008879.hg.1 | NM_002039 | 3,42385 | 0,00067932 | INCREASED |
| ZHX1 | TC0800012454.hg.1 | NM_001017926 | 3,42234 | 0,00131238 | INCREASED |
| C8orf88 | TC0800011074.hg.1 | NM_001190972 | 3,41199 | 0,00163862 | INCREASED |
| SAMD8 | TC1000008088.hg.1 | NM_001174156 | 3,39114 | 0,00838724 | INCREASED |
| CASP3 | TC0400012618.hg.1 | NM_004346 | 3,37851 | 0,00672809 | INCREASED |
| ATP8A1 | TC0400010558.hg.1 | NM_001105529 | 3,34201 | 0,00632668 | INCREASED |
| ZUFSP | TC0600012971.hg.1 | NM_145062 | 3,33584 | 0,00033748 | INCREASED |
| CLASP2 | TC0300010676.hg.1 | NM_001207044 | 3,31871 | 0,00567575 | INCREASED |
| RASSF8 | TC1200007120.hg.1 | NM_001164746 | 3,31616 | 0,00269183 | INCREASED |
| SYTL2 | TC1100011833.hg.1 | NM_001162951 | 3,31139 | 0,00331643 | INCREASED |
| ANKIB1 | TC0700008321.hg.1 | NM_019004 | 3,3044 | 0,00251399 | INCREASED |
| PHC3 | TC0300013090.hg.1 | NM_001308116 | 3,29346 | 0,00061912 | INCREASED |
| EIF3E | TC0800012451.hg.1 | NM_001568 | 3,26174 | 0,00824306 | INCREASED |
| RANBP6 | TC0900009479.hg.1 | NM_001243202 | 3,26038 | 0,00458243 | INCREASED |
| FAM92A1P1 | TC1500006993.hg.1 | ENST00000528023 | 3,2566 | 0,00362989 | INCREASED |
| KIAA0586 | TC1400007310.hg.1 | NM_001244189 | 3,24814 | 0,00514629 | INCREASED |
| FAM175A | TC0400011208.hg.1 | NM_139076 | 3,23878 | 0,00599553 | INCREASED |
| ZNF736P9Y | TC0Y00006978.hg.1 | ENST00000439586 | 3,2342 | 0,0006677 | INCREASED |
| RNF128 | TC0X00008054.hg.1 | NM_024539 | 3,1871 | 0,00725298 | INCREASED |
| CEP85L | TC0600013001.hg.1 | NM_001042475 | 3,1262 | 0,00784919 | INCREASED |
| TMEM14EP | TC0300012834.hg.1 | ENST00000408960 | 3,11842 | 0,00661193 | INCREASED |
| PTPRG | TC0300007645.hg.1 | NM_002841 | 3,10004 | 0,00651774 | INCREASED |
| NF1 | TC1700007478.hg.1 | NM_000267 | 3,09675 | 0,0071898 | INCREASED |
| ZNF468 | TC1900012026.hg.1 | NM_001008801 | 3,08083 | 0,00775993 | INCREASED |
| CTAGE12P | TC0900009781.hg.1 | ENST00000400348 | 3,07074 | 0,00356392 | INCREASED |
| BTF3L4P4 | TC0400012370.hg.1 | ENST00000506381 | 3,0524 | 0,00145108 | INCREASED |
| NBEAP3 | TC2200009183.hg.1 | OTTHUMT00000276575 | 3,02862 | 0,00729701 | INCREASED |
| SMC4 | TC0300009337.hg.1 | NM_001002800 | 3,02111 | 0,00962542 | INCREASED |
| PLCB4 | TC2000006674.hg.1 | NM_000933 | 3,00489 | 0,00452343 | INCREASED |
| RFX3 | TC0900009414.hg.1 | NM_001282116 | 2,98499 | 0,00041328 | INCREASED |
| ZNF182 | TC0X00011363.hg.1 | NM_001007088 | 2,98494 | 0,00753155 | INCREASED |
| AKR1C3 | TC1000012428.hg.1 | NM_001253908 | 2,98232 | 0,00054001 | INCREASED |
| ZNF33A | TC1000007333.hg.1 | NM_001278170 | 2,97271 | 0,00774079 | INCREASED |
| TDRD3 | TC1300007344.hg.1 | NM_001146070 | 2,97266 | 0,00438046 | INCREASED |
| SH3BGRL | TC0X00007744.hg.1 | NM_003022 | 2,95076 | 0,00839735 | INCREASED |
| PRR4 | TC1200012760.hg.1 | ENST00000541456 | 2,92935 | 0,0023256 | INCREASED |
| FNIP1 | TC0500013351.hg.1 | NM_001008738 | 2,92085 | 0,00242652 | INCREASED |
| RPS6KA3 | TC0X00009218.hg.1 | NM_004586 | 2,90943 | 0,00431136 | INCREASED |
| AP1AR | TC0400008427.hg.1 | NM_001128426 | 2,90523 | 0,00167956 | INCREASED |
| ANGPT2 | TC0800009418.hg.1 | NM_001118887 | 2,89461 | 0,0047812 | INCREASED |
| BHLHE41 | TC1200010182.hg.1 | NM_030762 | 2,89302 | 0,00614591 | INCREASED |
| HAUS6P3 | TC0700007574.hg.1 | ENST00000456660 | 2,87106 | 0,00823947 | INCREASED |
| KATNAL1 | TC1300008511.hg.1 | NM_001014380 | 2,85496 | 0,00916126 | INCREASED |
| DEK | TC0600010962.hg.1 | NM_001134709 | 2,83694 | 0,00735644 | INCREASED |
| USP9Y | TC0Y00006629.hg.1 | NM_004654 | 2,76598 | 0,00367869 | INCREASED |
| RBMS1P1 | TC1200011135.hg.1 | ENST00000417216 | 2,76438 | 0,00046897 | INCREASED |
| RAB3IP | TC1200012663.hg.1 | NM_001024647 | 2,76301 | 0,00979456 | INCREASED |
| RSRC1 | TC0300009306.hg.1 | NM_001271834 | 2,76223 | 0,00430631 | INCREASED |
| TVP23B | TC1700007138.hg.1 | NM_016078 | 2,73907 | 0,00298367 | INCREASED |
| SOCS6 | TC1800007620.hg.1 | NM_004232 | 2,73898 | 0,00513811 | INCREASED |
| VN1R108P | TC2000009901.hg.1 | ENST00000431580 | 2,73501 | 0,00992222 | INCREASED |
| ANKRD20A9P | TC1300010015.hg.1 | NR_027995 | 2,64425 | 0,00292146 | INCREASED |
| ZNF100 | TC1900010158.hg.1 | NM_173531 | 2,64191 | 0,00378391 | INCREASED |
| TRIM24 | TC0700009317.hg.1 | NM_003852 | 2,63659 | 0,00863906 | INCREASED |
| NAA30 | TC1400007285.hg.1 | NM_001011713 | 2,62584 | 0,0081127 | INCREASED |
| ZNF14 | TC1900010078.hg.1 | NM_021030 | 2,61534 | 0,00569833 | INCREASED |
| TTC3P1 | TC0X00010112.hg.1 | NR_030737 | 2,60839 | 0,00487376 | INCREASED |
| NF1P3 | TC2100006578.hg.1 | OTTHUMT00000157743 | 2,59213 | 0,00236969 | INCREASED |
| NREP | TC0500011705.hg.1 | NM_001142474 | 2,57784 | 0,00753979 | INCREASED |
| ETAA1 | TC0200007872.hg.1 | NM_019002 | 2,57026 | 0,0017613 | INCREASED |
| RAPGEF2 | TC0400012856.hg.1 | ENST00000503328 | 2,56964 | 0,0031445 | INCREASED |
| MFAP3L | TC0400012992.hg.1 | NM_001009554 | 2,56634 | 0,00110068 | INCREASED |
| RND3 | TC0200014597.hg.1 | NM_001254738 | 2,5648 | 0,00855748 | INCREASED |
| ARL5AP4 | TC2200006902.hg.1 | ENST00000430731 | 2,551 | 0,00817996 | INCREASED |
| DSE | TC0600014177.hg.1 | NM_001080976 | 2,54039 | 0,00138448 | INCREASED |
| KIAA1468 | TC1800007504.hg.1 | NM_020854 | 2,53578 | 0,0033904 | INCREASED |
| SEPT7P2 | TC0700010959.hg.1 | NR_024271 | 2,50729 | 0,00762312 | INCREASED |
| ZNF37A | TC1000007337.hg.1 | NM_001007094 | 2,48988 | 0,00531532 | INCREASED |
| TAS2R31 | TC1200012761.hg.1 | NM_176885 | 2,48101 | 0,00058661 | INCREASED |
| INO80D | TC0200015506.hg.1 | NM_017759 | 2,47935 | 0,00334343 | INCREASED |
| LEO1 | TC1500009446.hg.1 | NM_001286430 | 2,46183 | 0,00263144 | INCREASED |
| CENPJ | TC1300008371.hg.1 | NM_018451 | 2,46063 | 0,00395382 | INCREASED |
| TMED10P2 | TC0300008726.hg.1 | ENST00000474255 | 2,45764 | 0,00206686 | INCREASED |
| ATF7IP2 | TC1600006870.hg.1 | NM_001256160 | 2,45521 | 0,0092034 | INCREASED |
| GCOM1 | TC1500010744.hg.1 | NM_001018090 | 2,4478 | 0,00361335 | INCREASED |
| SERPINI1 | TC0300009412.hg.1 | NM_001122752 | 2,43849 | 0,00806965 | INCREASED |
| ZNF675 | TC1900011928.hg.1 | NM_138330 | 2,43386 | 9,18E-05 | INCREASED |
| L3HYPDH | TC1400010757.hg.1 | NM_144581 | 2,43279 | 0,00872078 | INCREASED |
| PRH1-PRR4 | TC1200012759.hg.1 | hsa_circ_0025461 | 2,43252 | 0,0002472 | INCREASED |
| PPP1R2 | TC0300013639.hg.1 | NM_001291504 | 2,41738 | 0,00770036 | INCREASED |
| KIAA1324L | TC0700011692.hg.1 | NM_001142749 | 2,41521 | 0,00231492 | INCREASED |
| VWA8P1 | TC0500010983.hg.1 | ENST00000510889 | 2,41044 | 0,00867998 | INCREASED |
| HACE1 | TC0600012702.hg.1 | NM_020771 | 2,40594 | 0,00921586 | INCREASED |
| PRRG1 | TC0X00011278.hg.1 | NM_000950 | 2,40485 | 0,00020176 | INCREASED |
| SP4 | TC0700006864.hg.1 | NM_003112 | 2,40335 | 0,00089717 | INCREASED |
| SCAND3P1 | TC1300006864.hg.1 | ENST00000439854 | 2,38425 | 0,00190642 | INCREASED |
| AFF4 | TC0500008656.hg.1 | hsa_circ_0001530 | 2,3518 | 0,00263465 | INCREASED |
| ARL5A | TC0200016739.hg.1 | ENST00000487818 | 2,34685 | 0,00400474 | INCREASED |
| FAM92A1 | TC0800008235.hg.1 | NM_001283034 | 2,34365 | 0,00182214 | INCREASED |
| ZNF286B | TC1700012371.hg.1 | NM_001145045 | 2,31856 | 0,00129016 | INCREASED |
| THUMPD2 | TC0200012335.hg.1 | NM_025264 | 2,31 | 0,00363296 | INCREASED |
| STIM2 | TC0400012782.hg.1 | NM_001169117 | 2,30925 | 0,00624762 | INCREASED |
| ZBTB33 | TC0X00008273.hg.1 | NM_001184742 | 2,2944 | 0,00049832 | INCREASED |
| NIPA1 | TC1500010691.hg.1 | NM_001142275 | 2,29065 | 0,00457272 | INCREASED |
| CEP63 | TC0300008888.hg.1 | NM_001042383 | 2,28078 | 0,00760019 | INCREASED |
| TMEFF1 | TC0900012154.hg.1 | NM_003692 | 2,2593 | 0,00880681 | INCREASED |
| EPC1 | TC1000010256.hg.1 | NM_001272004 | 2,24642 | 0,00442424 | INCREASED |
| NF1P5 | TC1800008172.hg.1 | ENST00000588287 | 2,24071 | 0,00751247 | INCREASED |
| BCO2 | TC1100013093.hg.1 | NM_001037290 | 2,23922 | 0,00915754 | INCREASED |
| RGS17 | TC0600013604.hg.1 | NM_012419 | 2,20256 | 0,00473054 | INCREASED |
| RPL36AP15 | TC0200012524.hg.1 | ENST00000444514 | 2,20115 | 0,00044182 | INCREASED |
| MTND4P32 | TC0X00007986.hg.1 | ENST00000437189 | 2,18987 | 0,00625443 | INCREASED |
| PCGF5 | TC1000008431.hg.1 | NM_001256549 | 2,18814 | 0,00605394 | INCREASED |
| YOD1 | TC0100017118.hg.1 | NM_001276320 | 2,16766 | 0,00083367 | INCREASED |
| ZNF248 | TC1000010362.hg.1 | NM_001267597 | 2,1649 | 0,0007227 | INCREASED |
| PPM1A | TC1400007354.hg.1 | NM_021003 | 2,1573 | 0,0039586 | INCREASED |
| CCBL2 | TC0100014848.hg.1 | NM_001008661 | 2,15263 | 0,00060467 | INCREASED |
| ZNF736P7Y | TC0Y00006976.hg.1 | ENST00000425158 | 2,14117 | 0,00881896 | INCREASED |
| LRRIQ1 | TC1200008344.hg.1 | NM_001079910 | 2,13325 | 0,00071608 | INCREASED |
| ST3GAL6 | TC0300013852.hg.1 | NM_001271142 | 2,11878 | 0,00157903 | INCREASED |
| FUNDC1 | TC0X00009498.hg.1 | NM_173794 | 2,11351 | 0,00249197 | INCREASED |
| FAM45A | TC1000009063.hg.1 | NM_001303111 | 2,10738 | 0,00544999 | INCREASED |
| ZBTB40-IT1 | TC0100007284.hg.1 | ENST00000438551.1 | 2,09101 | 0,00231852 | INCREASED |
| WWTR1 | TC0300012781.hg.1 | NM_001168278 | 2,08531 | 0,00418964 | INCREASED |
| LYPLAL1 | TC0100018344.hg.1 | NM_001300769 | 2,05113 | 0,00014501 | INCREASED |
| BMPR1APS1 | TC0600013140.hg.1 | ENST00000403332 | 2,03937 | 0,00877923 | INCREASED |
| SDCBPP3 | TC0X00009447.hg.1 | ENST00000419629 | 2,03778 | 0,00405594 | INCREASED |
| ZNF268 | TC1200012737.hg.1 | NM_001165881 | 2,0346 | 5,77E-05 | INCREASED |
| BNIP3P42 | TC0700007776.hg.1 | ENST00000454982 | 2,03263 | 0,00763136 | INCREASED |
| ARSK | TC0500008121.hg.1 | NM_198150 | 2,02229 | 0,00462883 | INCREASED |
| MTRF1L | TC0600013603.hg.1 | NM_001114184 | 2,01748 | 0,00388801 | INCREASED |
| CAST | TC0500013205.hg.1 | ENST00000510098 | 2,01129 | 0,00274865 | INCREASED |
| FAM213B | TC0100012543.hg.1 | hsa_circ_0000010 | -2,00807 | 0,00267546 | DECREASED |
| OR7E83P | TC0400006837.hg.1 | OTTHUMT00000359604 | -2,00866 | 0,00704697 | DECREASED |
| ZNF546 | TSUnmapped00000130.hg.1 | ENST00000625917 | -2,00924 | 0,00381873 | DECREASED |
| PSPH | TC0700011162.hg.1 | NM_004577 | -2,01002 | 0,00926553 | DECREASED |
| NOP56P1 | TC0600011272.hg.1 | ENST00000440030 | -2,01175 | 0,00717222 | DECREASED |
| CNPPD1 | TC0200015764.hg.1 | NM_015680 | -2,01411 | 0,00690717 | DECREASED |
| RRP7A | TC2200008881.hg.1 | NM_015703 | -2,01646 | 0,00087717 | DECREASED |
| GAMT | TC1900009155.hg.1 | NM_000156 | -2,02074 | 0,00441978 | DECREASED |
| FXYD5 | TC1900007839.hg.1 | NM_001164605 | -2,02254 | 0,00946476 | DECREASED |
| BRAT1 | TC0700010062.hg.1 | NM_152743 | -2,02395 | 0,00679692 | DECREASED |
| CD320 | TC1900011856.hg.1 | NM_001165895 | -2,0247 | 0,00893146 | DECREASED |
| MRPL46 | TC1500010921.hg.1 | NM_022163 | -2,03455 | 0,00827631 | DECREASED |
| DDX11 | TC1200007208.hg.1 | NM_001257144 | -2,03784 | 0,0008341 | DECREASED |
| LTBP3 | TC1100011234.hg.1 | NM_001130144 | -2,03907 | 0,00640536 | DECREASED |
| FLYWCH1 | TC1600006644.hg.1 | NM_001308068 | -2,04384 | 0,00339614 | DECREASED |
| TACR3 | TC0400011483.hg.1 | NM_001059 | -2,04843 | 0,00980489 | DECREASED |
| HIST1H2BD | TC0600007274.hg.1 | NM_021063 | -2,05135 | 0,00852959 | DECREASED |
| DDX12P | TC1200009868.hg.1 | NR_033399 | -2,05285 | 0,00545359 | DECREASED |
| KRT18P68 | TC0X00007100.hg.1 | ENST00000392293 | -2,05338 | 0,00362111 | DECREASED |
| PMM1 | TC2200008831.hg.1 | NM_002676 | -2,05755 | 0,00203665 | DECREASED |
| NT5C | TC1700011736.hg.1 | NM_001252377 | -2,05878 | 0,00906605 | DECREASED |
| IMP4 | TC0200009299.hg.1 | NM_033416 | -2,05887 | 0,0056459 | DECREASED |
| PDPK1 | TC1600006619.hg.1 | NM_001261816 | -2,05947 | 0,00413932 | DECREASED |
| MTHFS | TC1500010900.hg.1 | NM_001199758 | -2,06742 | 0,00392317 | DECREASED |
| KATNB1 | TC1600008011.hg.1 | NM_005886 | -2,07207 | 0,00299687 | DECREASED |
| LYSMD1 | TC0100015774.hg.1 | NM_001136543 | -2,07547 | 0,00397629 | DECREASED |
| PTDSS2 | TC1100006462.hg.1 | NM_030783 | -2,07784 | 0,00910995 | DECREASED |
| GART | TC2100008000.hg.1 | NM_000819 | -2,08375 | 0,00744571 | DECREASED |
| IFRD2 | TC0300013981.hg.1 | NM_006764 | -2,0886 | 0,00873731 | DECREASED |
| MGAT4B | TC0500013064.hg.1 | NM_014275 | -2,09632 | 0,00392611 | DECREASED |
| PFKL | TC2100007355.hg.1 | NM_001002021 | -2,0965 | 0,00523957 | DECREASED |
| ZNF674-AS1 | TC0X00007111.hg.1 | NR_015378 | -2,09736 | 0,00679326 | DECREASED |
| HOXB7 | TC1700012434.hg.1 | NM_004502 | -2,10238 | 0,00977403 | DECREASED |
| ABALON | TC2000007088.hg.1 | NR_131907 | -2,10297 | 0,00128251 | DECREASED |
| SORD2P | TC1500009271.hg.1 | ENST00000558556 | -2,11689 | 0,00693685 | DECREASED |
| BEX2 | TC0X00010409.hg.1 | NM_001168399 | -2,12243 | 0,00385886 | DECREASED |
| SORBS3 | TC0800012279.hg.1 | NM_001018003 | -2,1263 | 0,00276322 | DECREASED |
| RNF167 | TC1700006654.hg.1 | NM_015528 | -2,13638 | 0,00010122 | DECREASED |
| AAMP | TC0200015720.hg.1 | NM_001087 | -2,13781 | 0,00543488 | DECREASED |
| DYRK1B | TSUnmapped00000019.hg.1 | ENST00000625438 | -2,14076 | 0,00923712 | DECREASED |
| HGS | TC1700009189.hg.1 | NM_004712 | -2,14411 | 0,00603055 | DECREASED |
| ZNF358 | TC1900011652.hg.1 | NM_018083 | -2,15172 | 0,00984865 | DECREASED |
| SLC25A45 | TC1100011222.hg.1 | NM_001077241 | -2,15303 | 0,0034359 | DECREASED |
| GLTSCR2 | TC1900008433.hg.1 | NM_015710 | -2,1535 | 0,00854703 | DECREASED |
| ASS1P10 | TC0500008989.hg.1 | ENST00000507235 | -2,15483 | 0,00023651 | DECREASED |
| TBC1D24 | TC1600011329.hg.1 | NM_001199107 | -2,15596 | 0,00636478 | DECREASED |
| ELFN1 | TC0700006529.hg.1 | NM_001128636 | -2,15633 | 0,00900411 | DECREASED |
| TSTA3 | TC0800012147.hg.1 | NM_003313 | -2,15687 | 0,00453085 | DECREASED |
| DBNL | TC0700007367.hg.1 | NM_001014436 | -2,1569 | 0,00049139 | DECREASED |
| NRGN | TC1100009398.hg.1 | NM_001126181 | -2,16483 | 0,00669166 | DECREASED |
| PCBD2 | TC0500013230.hg.1 | NM_032151 | -2,16896 | 0,0007262 | DECREASED |
| GPANK1 | TC0600011443.hg.1 | NM_001199237 | -2,16896 | 0,00758712 | DECREASED |
| NOG | TC1700008350.hg.1 | NM_005450 | -2,17751 | 0,00869368 | DECREASED |
| HMBS | TSUnmapped00000307.hg.1 | ENST00000629150 | -2,18269 | 0,00421172 | DECREASED |
| SLC25A19 | TC1700011746.hg.1 | NM_001126121 | -2,18298 | 0,00870713 | DECREASED |
| SERPINA3 | TC1400010644.hg.1 | NM_001085 | -2,18581 | 0,00054132 | DECREASED |
| HIST1H2BI | TC0600007293.hg.1 | NM_003525 | -2,18768 | 0,00401916 | DECREASED |
| AP1M2 | TC1900009639.hg.1 | NM_001300887 | -2,18998 | 0,00323539 | DECREASED |
| DPRXP6 | TC0X00007017.hg.1 | OTTHUMT00000469421 | -2,19311 | 0,00582631 | DECREASED |
| DNMT1 | TC1900011860.hg.1 | NM_001130823 | -2,20126 | 0,00534092 | DECREASED |
| SURF2 | TSUnmapped00000645.hg.1 | ENST00000630633 | -2,20355 | 0,00109006 | DECREASED |
| OR6M1 | TC1100012643.hg.1 | NM_001005325 | -2,21527 | 0,00730715 | DECREASED |
| AJUBA | TC1400010715.hg.1 | NM_001289097 | -2,21578 | 0,00199431 | DECREASED |
| ASS1P5 | TC0X00008200.hg.1 | ENST00000435496 | -2,21713 | 0,00357271 | DECREASED |
| MYBBP1A | TC1700009515.hg.1 | NM_001105538 | -2,22644 | 0,00505965 | DECREASED |
| AXL | TC1900008143.hg.1 | NM_001278599 | -2,24488 | 0,00767556 | DECREASED |
| WDR73 | TC1500010290.hg.1 | NM_032856 | -2,24845 | 0,0051304 | DECREASED |
| HIST2H2AC | TC0100009880.hg.1 | NM_003517 | -2,25186 | 0,00090984 | DECREASED |
| JUN | TC0100014349.hg.1 | NM_002228 | -2,25396 | 0,0092463 | DECREASED |
| BCL2L1 | TC2000008815.hg.1 | NM_001191 | -2,26984 | 0,00601934 | DECREASED |
| APEX1 | TC1400006519.hg.1 | NM_001244249 | -2,28963 | 0,00293408 | DECREASED |
| SDC4 | TC2000009218.hg.1 | NM_002999 | -2,31355 | 0,00786861 | DECREASED |
| OIP5 | TC1500009148.hg.1 | NM_007280 | -2,31866 | 0,00435528 | DECREASED |
| SCARNA2 | TC0100009340.hg.1 | NR_003023 | -2,32842 | 0,0025563 | DECREASED |
| HIST1H4C | TC0600007268.hg.1 | NM_003542 | -2,3313 | 0,00614168 | DECREASED |
| ASF1B | TC1900009807.hg.1 | NM_018154 | -2,34372 | 0,00253209 | DECREASED |
| COL17A1 | TC1000011740.hg.1 | NM_000494 | -2,36656 | 0,00263529 | DECREASED |
| ZNF580 | TC1900011804.hg.1 | NM_001163423 | -2,36907 | 0,00950189 | DECREASED |
| KRT80 | TC1200010740.hg.1 | NM_001081492 | -2,37968 | 0,00262515 | DECREASED |
| ALG1L2 | TC0300013866.hg.1 | NM_001136152 | -2,38974 | 0,00713761 | DECREASED |
| HMBS | TC1100009238.hg.1 | NM_000190 | -2,4007 | 0,00704931 | DECREASED |
| HIST1H4E | TC0600007284.hg.1 | NM_003545 | -2,40439 | 0,00898441 | DECREASED |
| FAM83H | TC0800012160.hg.1 | NM_198488 | -2,40735 | 0,00531356 | DECREASED |
| KCNE3 | TC1100011614.hg.1 | NM_005472 | -2,41448 | 0,00529523 | DECREASED |
| SMPDL3B | TC0100007542.hg.1 | NM_001009568 | -2,43836 | 0,00220726 | DECREASED |
| FOSL1 | TC1100011259.hg.1 | NM_001300844 | -2,45432 | 0,00629018 | DECREASED |
| RAP1GAP2 | TC1700006569.hg.1 | NM_001100398 | -2,47447 | 0,00473872 | DECREASED |
| SOX9 | TC1700008794.hg.1 | NM_000346 | -2,52021 | 0,00410343 | DECREASED |
| HIST3H2BB | TC0100011871.hg.1 | NM_175055 | -2,53162 | 0,00845645 | DECREASED |
| BOP1 | TC0800012202.hg.1 | NM_015201 | -2,54258 | 0,00421823 | DECREASED |
| S100A4 | TC0100015866.hg.1 | NM_002961 | -2,54515 | 0,00269344 | DECREASED |
| SUV39H1 | TC0X00007200.hg.1 | NM_001282166 | -2,56982 | 0,00318277 | DECREASED |
| darvee | TC2200008754.hg.1 | darvee.aAug10 | -2,57954 | 0,00064497 | DECREASED |
| SLC7A15P | TC0200006888.hg.1 | ENST00000424028 | -2,58218 | 0,0096151 | DECREASED |
| EIF4EBP1 | TC0800007316.hg.1 | NM_004095 | -2,58996 | 0,00172234 | DECREASED |
| TMEM97 | TC1700007360.hg.1 | NM_014573 | -2,5925 | 0,0006756 | DECREASED |
| KRT7 | TC1200012634.hg.1 | NM_005556 | -2,59486 | 0,00473068 | DECREASED |
| HIST1H4K | TC0600011227.hg.1 | NM_003541 | -2,60029 | 0,00169202 | DECREASED |
| SLC19A1 | TC2100008424.hg.1 | NM_001205206 | -2,60268 | 0,00761254 | DECREASED |
| BDH1 | TC0300013751.hg.1 | NM_004051 | -2,61022 | 0,00192099 | DECREASED |
| DCTPP1 | TC1600009966.hg.1 | NM_024096 | -2,67625 | 0,00233169 | DECREASED |
| LTBP4 | TC1900008113.hg.1 | NM_001042544 | -2,68359 | 0,00399619 | DECREASED |
| HIST1H1C | TC0600011127.hg.1 | NM_005319 | -2,69079 | 0,00984645 | DECREASED |
| FOXRED2 | TC2200008610.hg.1 | NM_001102371 | -2,70046 | 0,00491183 | DECREASED |
| ERP29 | TC1200008900.hg.1 | NM_001034025 | -2,72149 | 0,00202809 | DECREASED |
| HIST1H2BF | TC0600007282.hg.1 | NM_003522 | -2,72627 | 0,00962189 | DECREASED |
| GSG2 | TC1700006592.hg.1 | NM_031965 | -2,73953 | 0,00858177 | DECREASED |
| SESN2 | TC0100007552.hg.1 | NM_031459 | -2,78148 | 0,00052617 | DECREASED |
| EXOSC5 | TC1900010746.hg.1 | NM_020158 | -2,7952 | 0,00177764 | DECREASED |
| PYCR1 | TC1700012484.hg.1 | NM_001282279 | -2,80445 | 0,0004172 | DECREASED |
| ZBTB3 | TC1100011097.hg.1 | NM_024784 | -2,81848 | 0,00301473 | DECREASED |
| HIST1H2AB | TC0600011125.hg.1 | NM_003513 | -2,83738 | 0,00293176 | DECREASED |
| ATF4P3 | TC1700011789.hg.1 | OTTHUMT00000255904 | -2,8388 | 0,00273346 | DECREASED |
| CDCA5 | TC1100013184.hg.1 | NM_080668 | -2,84033 | 0,00162507 | DECREASED |
| TP73 | TC0100006620.hg.1 | NM_001126240 | -2,86239 | 0,00812779 | DECREASED |
| URM1 | TC0900012176.hg.1 | NM_001135947 | -2,86708 | 0,00238549 | DECREASED |
| DDX11L2 | TC0200013936.hg.1 | NR_024004 | -2,90276 | 0,00873182 | DECREASED |
| ANO1 | TC1100008257.hg.1 | NM_018043 | -2,93976 | 0,00856849 | DECREASED |
| CDK4 | TC1200010977.hg.1 | NM_000075 | -2,9759 | 0,00752814 | DECREASED |
| MYC | TC0800008845.hg.1 | NM_002467 | -3,00718 | 0,00249873 | DECREASED |
| HIST1H2APS4 | TC0600011143.hg.1 | OTTHUMT00000040101 | -3,01317 | 0,00268124 | DECREASED |
| JDP2 | TC1400007710.hg.1 | NM_001135047 | -3,03968 | 0,00117004 | DECREASED |
| HIST1H1E | TC0600007273.hg.1 | NM_005321 | -3,04421 | 0,00168292 | DECREASED |
| TP53 | TC1700009651.hg.1 | NM_000546 | -3,04873 | 0,00540116 | DECREASED |
| SLC43A3 | TC1100013165.hg.1 | NM_001278201 | -3,09128 | 0,00555041 | DECREASED |
| NAPRT | TC0800012143.hg.1 | NM_001286829 | -3,09478 | 0,00749812 | DECREASED |
| CHRNA3 | TC1500010151.hg.1 | NM_000743 | -3,09706 | 0,00121687 | DECREASED |
| ATF4 | TC2200007406.hg.1 | NM_001675 | -3,15125 | 0,00173844 | DECREASED |
| CHAF1B | TC2100008508.hg.1 | NM_005441 | -3,19253 | 0,00334421 | DECREASED |
| RBCK1 | TC2000006446.hg.1 | NM_006462 | -3,22268 | 0,00838665 | DECREASED |
| HIST2H2AB | TC0100015711.hg.1 | NM_175065 | -3,24351 | 0,00219128 | DECREASED |
| HIST2H2AA3 | TC0100009876.hg.1 | NM_003516 | -3,29618 | 0,00611432 | DECREASED |
| HIST1H2AI | TC0600007374.hg.1 | NM_003509 | -3,36542 | 0,0030428 | DECREASED |
| HIST1H2AL | TC0600007380.hg.1 | NM_003511 | -3,36956 | 0,00796785 | DECREASED |
| HIST1H2AM | TC0600011235.hg.1 | NM_003514 | -3,40248 | 0,00698694 | DECREASED |
| CHAF1A | TC1900006691.hg.1 | NM_005483 | -3,41366 | 0,0010807 | DECREASED |
| HIST1H2BJ | TC0600011184.hg.1 | NM_021058 | -3,51384 | 0,00235836 | DECREASED |
| PSMC3IP | TC1700010731.hg.1 | NM_001256014 | -3,59375 | 0,00300524 | DECREASED |
| CDT1 | TC1600008845.hg.1 | NM_030928 | -3,61681 | 0,00071494 | DECREASED |
| E2F1 | TC2000008894.hg.1 | NM_005225 | -3,63746 | 0,00398582 | DECREASED |
| FAM27E3 | TC0900010311.hg.1 | NR_103833 | -3,67716 | 0,00120257 | DECREASED |
| FEN1 | TC1100013021.hg.1 | NM_004111 | -3,76284 | 0,0028945 | DECREASED |
| HIST1H2AG | TC0600014083.hg.1 | NM_021064 | -3,78272 | 0,00058466 | DECREASED |
| HIST1H2AJ | TC0600011225.hg.1 | NM_021066 | -3,78372 | 0,00289287 | DECREASED |
| HIST2H4B | TC0100009870.hg.1 | NM_001034077 | -3,85811 | 0,00209294 | DECREASED |
| HIST2H4A | TC0100015707.hg.1 | NM_003548 | -3,96576 | 0,00063893 | DECREASED |
| HIST1H2AK | TC0600011228.hg.1 | NM_003510 | -3,97073 | 0,00396792 | DECREASED |
| GINS2 | TC1600011574.hg.1 | NM_016095 | -3,99818 | 0,00943149 | DECREASED |
| TLCD1 | TC1700010209.hg.1 | NM_001160407 | -4,27686 | 0,00902008 | DECREASED |
| MCM5 | TC2200007206.hg.1 | NM_006739 | -5,02388 | 0,00773444 | DECREASED |
| HIST2H3A | TC0100015701.hg.1 | NM_001005464 | -5,2075 | 0,00227736 | DECREASED |
| HIST2H3A | TC0100009877.hg.1 | ENST00000403683 | -5,28039 | 0,00200141 | DECREASED |
| CHAC1 | TC1500010723.hg.1 | NM_001142776 | -5,85796 | 0,00664003 | DECREASED |
